# Structural Characterization of Heat Shock Protein 90β and Molecular Interactions with Geldanamycin and Ritonavir: A Computational Study

**DOI:** 10.3390/ijms25168782

**Published:** 2024-08-12

**Authors:** Carlyle Ribeiro Lima, Deborah Antunes, Ernesto Caffarena, Nicolas Carels

**Affiliations:** 1 Laboratory of Biological System Modeling, Centro de Desenvolvimento Tecnológico em Saúde (CDTS), Fundação Oswaldo Cruz (FIOCRUZ), Rio de Janeiro 21040-900, Brazil; 2 Laboratório de Genômica Aplicada e Bioinovações, Instituto Oswaldo Cruz, Fundação Oswaldo Cruz (FIOCRUZ), Rio de Janeiro 21040-900, Brazil; deborah.santos@fiocruz.br; 3 Grupo de Biofísica Computacional e Modelagem Molecular, Programa de Computação Científica (PROCC), Fundação Oswaldo Cruz (FIOCRUZ), Rio de Janeiro 21040-900, Brazil; ernesto.caffarena@fiocruz.br

**Keywords:** cancer, Hsp9β, ritonavir, geldanamycin, drug repurposing, chemical ligations, molecular docking, molecular dynamics

## Abstract

Drug repositioning is an important therapeutic strategy for treating breast cancer. Hsp90β chaperone is an attractive target for inhibiting cell progression. Its structure has a disordered and flexible linker region between the N-terminal and central domains. Geldanamycin was the first Hsp90β inhibitor to interact specifically at the N-terminal site. Owing to the toxicity of geldanamycin, we investigated the repositioning of ritonavir as an Hsp90β inhibitor, taking advantage of its proven efficacy against cancer. In this study, we used molecular modeling techniques to analyze the contribution of the Hsp90β linker region to the flexibility and interaction between the ligands geldanamycin, ritonavir, and Hsp90β. Our findings indicate that the linker region is responsible for the fluctuation and overall protein motion without disturbing the interaction between the inhibitors and the N-terminus. We also found that ritonavir established similar interactions with the substrate ATP triphosphate, filling the same pharmacophore zone.

## 1. Introduction

Cancer is an emerging concern worldwide [1]. Among solid tumors, breast cancer (BC) is a significant health challenge for women, with almost 2.1 million new cases diagnosed annually worldwide, causing approximately 600,000 deaths each year [2]. BC is the leading cause of cancer-related death in women in Brazil, with approximately 57,000 new cases annually in a population of approximately 212 million (https://www.worldometers.info/world-population/brazil-population/, accessed on 20 May 2024), which corresponds to an estimated incidence of 56 cases per 100,000 women [3].

Using a triple-negative BC cell line (MDA-MB-231), it was shown that the selective inhibition through siRNA of the mRNA for the top five most connected hub proteins among the upregulated genes led to a significant decrease in cell proliferation, growth migration, and invasion, as well as to the induction of cell death [4]. These five genes were already reported to be up-regulated in breast cancer [5,6,7,8,9,10,11,12,13], and they encode for HSP90AB1 (the beta member of the HSP90 family of heat shock proteins—HSP), VIM (vimentin, a type III mesenchymal intermediate filament), CSNK2B (casein kinase 2β), YWHAB (a member of the 14-3-3 family of proteins), and TK1 (thymidine kinase 1).

Previous studies [4,14] have suggested the possibility of an individual therapy approach based on the molar diagnosis of hub targets upregulated in tumors (theranostic). The fastest way to apply this methodology to patients in a translational medicine framework is through drug repositioning. In this context, new specific inhibitors of malignant cells, differing from the existing cytotoxic drugs in their mechanism of action, are desired to alleviate symptoms and improve the survival rate of patients [15].

To assess drug repositioning, we employed molecular modeling methodologies to meticulously curate a computational three-dimensional (3D) representation of HSP90AB1 (Hsp90β), enabling a comparative evaluation of the binding affinities of geldanamycin (GDM) and ritonavir (RIT) towards this protein. Hsp90β appears to be a good target, mainly because it participates in stabilizing and transporting various proteins that extensively affect most biological functions [16].

Furthermore, its overexpression in a wide range of cancer types and the joint actions of stabilizing proteins that promote essential functions of tumor cells make proteins from the HSP family (in particular Hsp90β) key targets for tumor therapies [17].

GDM is an Hsp90 inhibitor that exhibits potent antitumor activity by targeting the N-terminal ATP-binding pocket of Hsp90 [18], which inhibits Hsp90 ATPase activity and affects dissociation of the mature chaperone Hsp90 complex. This process leads to the degradation of Hsp90 client proteins by E3 ligase in the ubiquitin–proteasome pathway [19]. The mechanisms involved in the proteasomal degradation of Hsp90 client proteins and destabilization of Hsp90 complexes are relatively well understood, but little is known about the effects of GDM on the modulation of Hsp90 genes at the transcriptional level [20].

In contrast, RIT, an HIV protease inhibitor, acts on HSP90 family members [21], although its specific target in MDA-MB-231 cells remains unknown. This drug is bioavailable, and its toxicity profile enables daily dosing [22]. Previous studies have revealed that RIT and its derivatives [23] show good activity in inhibiting CYP P450 3A4, which is closely related to breast cancer evolution [24,25]. Here, we propose a theoretical strategy to investigate RIT activity using an integrated approach involving molecular modeling, molecular docking, molecular dynamics (MD), and free energy analyses. Thus, we introduced a computationally derived three-dimensional (3D) conformation of the N-terminal (NTD) to the central domain (CD) linkage, followed by an analysis involving molecular dynamics (MD) simulations to examine the dynamic attributes and stability of complexes formed by Hsp90β with ATP, GDM, and RIT. Moreover, pivotal functional motifs were delineated by modeling chemical ligations. We found that ritonavir has a lower affinity for Hsp90β than ATP and GDM because of its higher rotamer number. However, the affinity difference is small, and it remains in the active site, which makes it a potential specific inhibitor of Hsp90β.

## 2. Results

### 2.1. Comparative Modeling

The sequence P08238 of Hsp90β exhibits a high degree of identity (99%) and similarity (100%) to the template (PDB ID: 5FWK), determined by cryo-EM with a resolution of 3.90 Å [26]. Hsp90β is segmented into four regions: (i) N-terminal domain (NTD: 1–201 AA), (ii) disordered loop (DL: 202–301 AA), (iii) central domain (CD: 302–504 AA), and (iv) C-terminal domain (CTD: 505–724 AA). Visual observations showed that the predicted local-distance difference (pLDDT) by AlphaFold2 converged with the disordered regions predicted by both PrDos and IUPred. More precisely, this convergence can be seen in the disordered alignment 1D (Figure S1).

**Figure 1:**
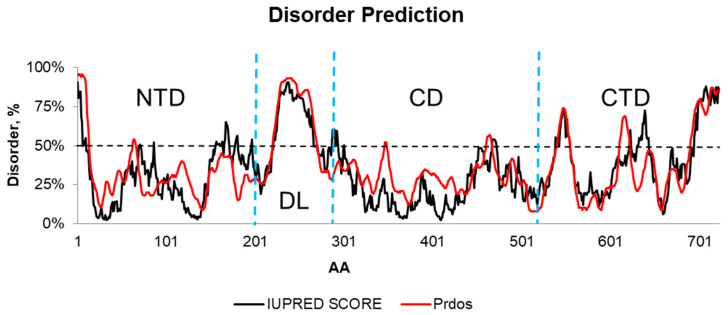
Profile of order levels of the Hsp90β 3D structure. NTD (N-terminal), DL (disordered loop), CD (central domain), and CTD (C-terminal) regions extend between 1–201, 202–301, 302–504, and 505–724 amino acids, respectively. Regions > 50% (horizontal dotted line) were considered disordered. The dotted blue lines represent the boundaries between the NTD, DL, CD, and CTD.

However, it is noteworthy that the predicted local-distance difference (pLDDT), a quality confidence metric utilized by AlphaFold2, is consistently aligned with the disordered regions of the NTD, DL, and CTD, as predicted by the PrDos and IUPred servers (Figure S1). Consequently, we decided to exclude these disordered regions from the AlphaFold2 model in the NTD and CTD regions only when their PrDos and IUPred scores surpassed the 50% threshold (Figure 1). This strategic adjustment aimed to reconcile potential disparities between disorder predictions and the pLDDT metric, ensuring a more accurate representation of the structural dynamics of the Hsp90β protein.

During the fourteenth iteration of the Critical Assessment of Structure Prediction (CASP), the MoldFold8 method emerged as the highest-ranking predictor for assessing protein model fidelity. This evaluation encompassed criteria such as (i) confidence score and *p*-value, and (ii) the overarching model quality score. Models with confidence scores and *p*-values below 0.001 were deemed accurate, while global model quality scores exceeding 0.4 indicated heightened faithfulness to native structures [27]. In our context, the selected model (Model 5) showed a confidence score and *p*-value of 8.20 × 10^−5^ and 8.78 × 10^−5^, respectively, and an overall model quality score of 0.5708. TM-score quality metrics aim to analyze the topological similarity of the model with the reference structure used as a mold. In this analysis, two factors were considered: (i) the folding of the protein, which is sensitive to local structural variations, and (ii) the normalization of the distances between the structures, regardless of their lengths. When we compared Model 5 with its respective AlphaFold2 and PDB templates, we found that Model 5 had TM scores of 0.974 and 0.983 and RMSD values of 1.548 Å and 1.285 Å, respectively.

**Figure 2:**
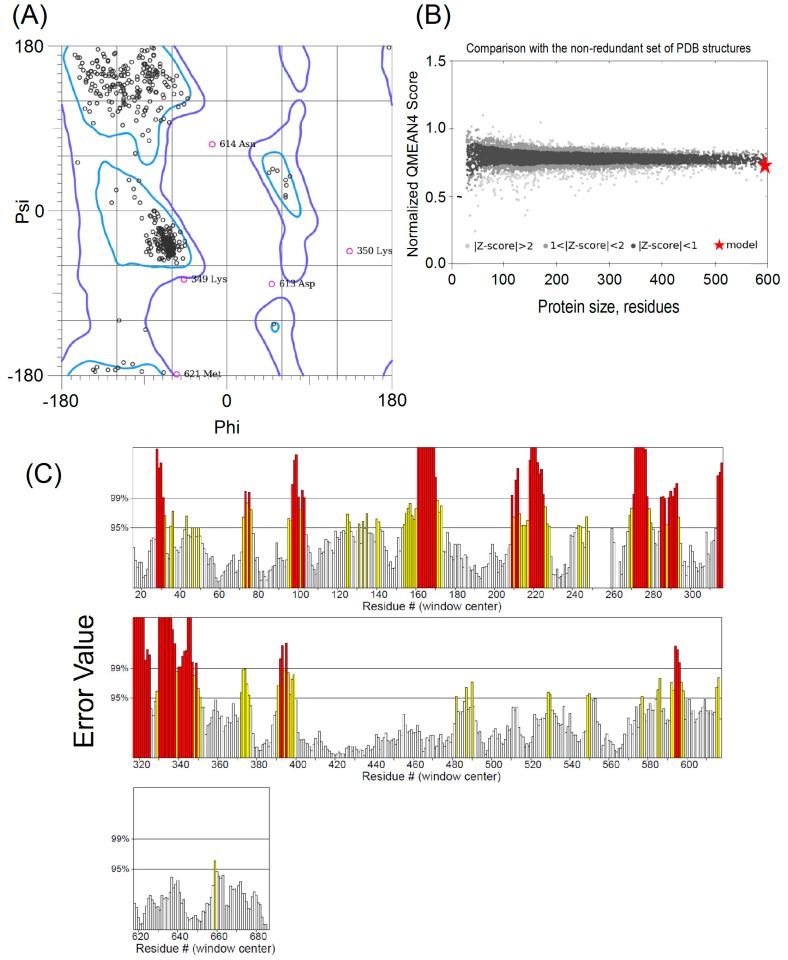
Quality assessment of Model 5 for Hsp90β. (**A**) Ramachandran graph of Model 5 with 96.75% favorable angles; (**B**) Qmean raw; (**C**) ERRAT plot for Hsp90β. Error-values were plotted as a function of the position of the sliding window of nine residues. Regions of the structure that can be rejected at 95% and 99% confidence levels are shown as yellow and red bars, respectively. White bars indicate regions in which protein folding can be considered reliable.

According to Molprobity, Model 5 exhibited a notable percentage of favorable angles, calculated at 96.75%. This server analyzes the stereochemistry of the models by considering the correct torsion of angles ϕ and ψ and the location of these residues in the favorable regions of the Ramachandran graph. Comparatively, the corresponding AlphaFold2 and PDB templates were 94.92% and 96.78%, respectively (Figure 2A).

Qmean employs raw scores and statistical parameters, yielding pseudo-energies with negative values, implying energetically favorable conformations [28]. The Qmean scores span the interval between 0 and 1, with proximity to 1 representing a heightened model quality. In Model 5, the Qmean score values stood at 0.77 ± 0.050; collectively, the models exhibited an average Qmean score of 0.71 ± 0.004 (Figure 2B).

The ERRAT server was used to analyze the quality of the models. Model 5 presented the best overall quality factor for unbound atomic interactions, with a score of 73.130. On a scale of 0 to 100, the acceptable range for a high-quality model was >50 (all other models had scores above 50, with a mean of 73.499 ± 1.574) (Figure 2C).

Model 5 was selected as the most qualified for further analyses; details of the scores obtained with each method for the other models are documented in Table S1.

After refining Model 5 with the ModRefine server, we compared it to the original model, revealing an RMSD value of 2.3 Å, primarily attributed to variations in the N-terminal domain (NTD) (Figure S2A). The refined model exhibited a significantly negative electrostatic profile, emphasizing the NTD and DL regions, whereas the CD and CTD regions alternated between positively and neutrally charged states (Figure S2B). In contrast, the model displayed predominantly hydrophilic hydropathy, particularly in the DL region, whereas the N-terminal domain (Figure S2C) exhibited hydrophobic characteristics.

### 2.2. Molecular Dynamics of the Hsp90β Model

To better understand the interactions that occur in the Hsp90β complexes with GDM and RIT, we used MD simulations of the Hsp90β complex with ATP as a positive control. The MD simulations encompassed a disordered DL region. However, the analyses were performed considering two situations: (i) considering the DL region and (ii) disregarding the DL region. To better understand the interactions taking place in the complexes of Hsp90β with GDM or RIT, we used an MD simulation of the Hsp90β complex with ATP as a positive control. The MD simulations encompassed a disordered DL region.

**Figure 3:**
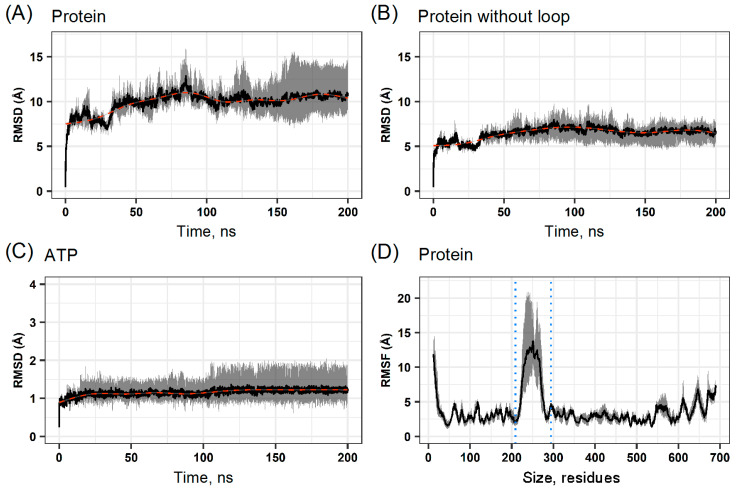
Root mean square deviation and fluctuation of Hsp90β complexed with ATP. (**A**) RMSD of Hsp90β with DL domain (loop). (**B**) RMSD of Hsp90β, disregarding the DL domain. (**C**) RMSD of ATP. (**D**) RMSF of Hsp90β in the DL region is highlighted (blue dots interval). The punctuated red lines represent smoothed averages (black) of fluctuations (gray).

When we analyzed Hsp90β by considering the DL, we found that the RMSD reached a maximum of 15.0 Å but stabilized at 10 Å RMSD ± 2.05 Å (Figure 3A).

On the other hand, the protein without the DL region (responsible for the noise induced by the loop movement) showed a maximum deviation of 10 Å and stabilized at 6 Å ± 1.23 Å RMSD from halfway through the simulation (Figure 3B). The RMSD of the ATP substrate complexed at the active site of Hsp90β, located in the N-terminal region, did not exceed 2.0 Å ± 0.37 Å and remained stable throughout the simulation (Figure 3C). Using root mean square fluctuation (RMSF) analysis, we also found that the more considerable mobility was in the DL region, with peaks up to 20.0 Å ± 2.82 Å (Figure 3D).

**Figure 4:**
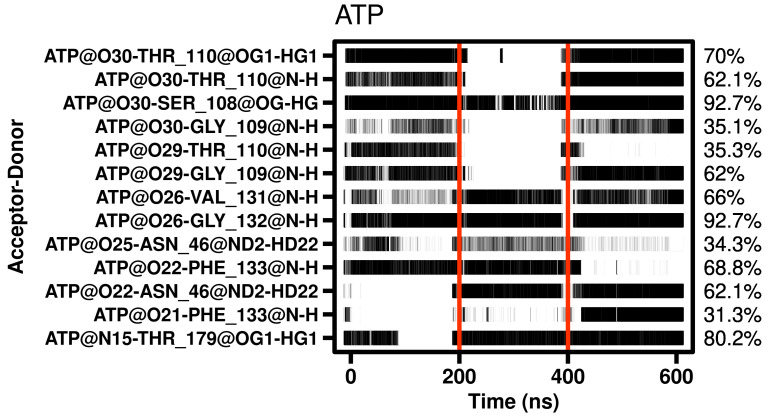
Hydrogen bonds identified by MD simulation of the Hsp90–ATP complex as a function of time.

During the simulation, several intermolecular polar and hydrophobic contacts were observed to form, break, and reform the protein complexes. H-bond interactions were assessed across all simulation replicates, encompassing a cumulative duration of 600 ns. Only the interactions exhibiting a sustained prevalence of at least 30% of the total simulated time were considered. The data revealed that in the Hsp90β–ATP complex, interactions were established with Asn46, Ser108, Gly109, Thr110, Phe122, Val131, Gly132, and Thr179 (at least 60% of the simulation time) (Figure 4).

Among the four clusters identified in the MD simulations, the most representative, determined using concatenated trajectories, accounted for 95%. The RMSD for this cluster, when compared to the MD of the initial structure of Hsp90β complexed with ATP was 3.54 Å, which represents an RMSD increase of 3 Å compared to the difference between the minimized structure and the best cluster. Notably, upon analyzing the interactions formed by ATP within the active site, considering the most representative cluster, a significant increase in hydrogen bonds was observed compared with the MD of the initial structure.

Comparison of the initial structure to the most representative cluster (Figure S3A,B) revealed the retention of crucial H-bond interactions, including those with Asn46, Ser108, Gly109, Thr110, Val131, and Gly132, observed in the MD simulation with the original structure of Hsp90β complexed with ATP.

Additionally, interactions involving (i) H-bonds and pi-alkyls with Ala50 and Met93, (ii) attractive charges of ATP’s triphosphate tail with Arg392, and (iii) metal acceptors with Mg^2+^ were also consistently preserved within this cluster. Further insights into the interatomic distances can be found in Tables S2 and S3.

### 2.3. Molecular Docking and Ligand Interactions

**Figure 5:**
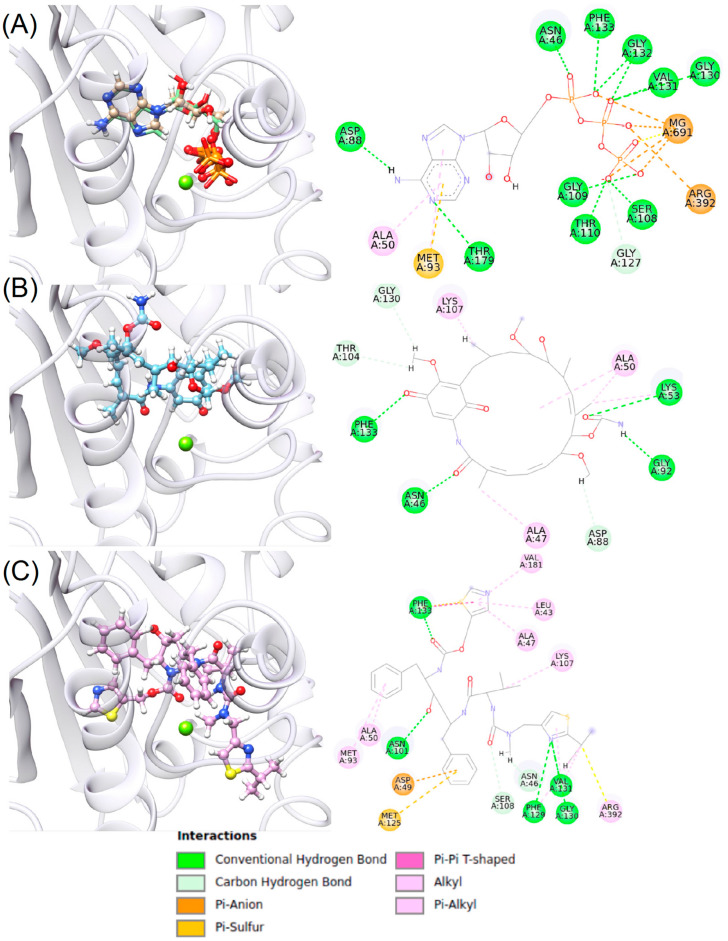
ATP redocking and docking of GDM and RIT ligands in the active site of Hsp90β. (**A**) ATP redocking, (**B**) GDM docking, and (**C**) RIT docking. Left: 3D docking of ligands in the best cluster of the Hsp90β active site; right: 2D interaction map of ligands in the most relevant Hsp90β cluster.

First, the docking pose was validated by redocking ATP onto its 3D structure in the most relevant Hsp90β cluster. Except for triphosphate tail rotamers, ATP redocking produced the same pose in the same binding pocket of the crystal model with a DOCK6 score of −148.34 kcal/mol and an RMSD of 0.3 Å as shown on the 2D mapping of Figure 5A—left.

After ATP redocking, GDM and RIT were docked in the active site with DOCK6 scores of −35.67 kcal/mol and −72.17 kcal/mol, respectively (Figure 5B,C—left). The reduced DOCK6 score of GDM and RIT compared to ATP is because ATP contains more oxygen than the other two ligands do. GDM and RIT ligands interact with (i) Asn46, Lys53, Gly92, and Phe133, and (ii) Asn101, Phe129, Gly130, Val131, and Phe133, respectively. All the ligands interacted with Thr110 and the Mg^2+^ ion; however, only GDM interacted with Gly130, Gly132, and Phe133, like ATP. In contrast, RIT interacted similarly to ATP only with Phe129 (Figure 5B,C—right).

However, RIT showed more types of interactions, such as (i) pi-alkyl, (ii) pi-anion, (iii) pi-sulfur, and (iv) Pi-Pi T-shared, with more amino acids than GDM, which suggests that RIT may have more affinity than GDM (Tables S4 and S5).

To better understand the chemical environment of Hsp90β–ligand complexes, we analyzed 3D interactions at the active site. In each model of chemical constituents, (i) the red arrows indicate the H-bond acceptor, (ii) green arrows indicate the H-bond donor, (iii) yellow spheres represent hydrophobic residues, (iv) blue rings represent aromatic rings, and (v) blue spheres represent positive ionizable residues.

**Figure 6:**
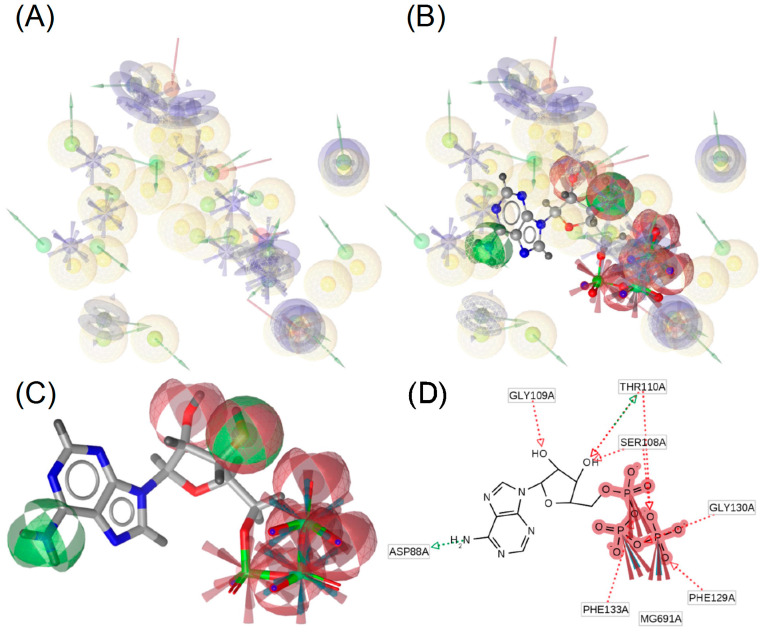
3D interaction analysis of the best cluster of Hsp90β–ATP with its foci in the N-terminal region. (**A**) Regions that interact with the active site of the best cluster of Hsp90β. (**B**) Interaction of ATP with the active site. (**C**) Regions of interaction of ATP: red—acceptor H-bond; green—donor H-bond. (**D**) 2D representation of ATP interactions. Yellow spheres represent hydrophobic interactions, blue spheres represent aromatic rings, red arrows indicate H-bond acceptors, and green arrows indicate the presence of H-bond donors.

3D interaction analysis of the Hsp90β active site revealed a predominance of hydrophobic regions (Figure 6A) close to the adenosine group of ATP (Figure 6B). Interestingly, the triphosphate tail of ATP occupies an Hsp90β region where aromatic rings are predominant; however, the triphosphate tail of ATP interacts with ionizable groups (NH_3_), such as Thr110, Phe129, and Phe133, which act as H donors (Figure 6C,D).

GDM and RIT ligands occupy the same region as the triphosphate tail of ATP; however, only RIT interacts with Phe129 and Gly130 as ATP does. This suggests that these AA are relevant to the interaction with the protein (Figures S4 and S5).

### 2.4. Molecular Dynamics of Geldanamycin and Ritonavir

The stability of Hsp90β complexed with GDM (protein + ligand) throughout the MD simulation (6 Å ± 2.28 Å of RMSD) was larger than with RIT (8 Å ± 2.28 Å of RMSD). MD stability was achieved after half the simulation time (Figure S6). When we did not consider the noise caused by the LD region, we found smaller deviations of 5 Å ± 0.97 and 7 Å ± 1.49 for GDM and RIT, respectively (Figure S7). The RMSD of GDM and RIT at the active site did not exceed 3 Å. RMSD values of 1.5 Å ± 0.19 and 2.8 Å ± 0.5 for GDM and RIT, respectively, were found after 100 ns of simulation, which remained stable until the end of MD (Figures S6 and S7).

The fluctuation of Hsp90β in the presence of GDM and RIT followed a behavior similar to that observed when complexed with ATP; that is, only the region between AA 200 and 300 showed high fluctuation with an RMSF as large as 15 Å ± 1.77 for GDM and ± 2.12 RIT, whereas the RMSF of the NTD and CD regions did not exceed 5 Å (Figure 6D and Figure S6).

To analyze the H-bonds formed by GDM and RIT over time, we applied a cutoff of 10%; that is, only H-bonds with a frequency greater than 10% in the total simulation were considered as relevant interactions. We observed that the most frequent interactions made by GDM (%) were with Gly92 and Asn101, whereas those made by RIT were with Asn101 and Gly132 (Figures S8 and S9).

Furthermore, we identified the best clusters for the Hsp90β complexes with GDM and RIT. These clusters accounted for 61.84% and 52.80% of the MD, respectively. The most relevant Hsp90β–GDM cluster (Figure S10) showed H-bonds with Met93, Asn101, Met125, and Gly132 while RIT showed H-bonds with Asn101, Gly130, Gly132, and Val131 (Figure S11).

### 2.5. Free Energy

Δ*G_bind_* for ATP, GDM, and RIT was derived from the values of dissociation constants (*K_d_*) published in the literature: (i) 400 nM for ATP [29], (ii) 1215 nM for GDM [30], and (iii) 7800 nM for RIT [15]), which correspond to −9.07, −8.39, −7.24 kcal/mol for ATP, GDM, RIT, respectively, according to the method described by Lima et al. [31]. When calculating the MMPBSA to determine the Δ*G_bind_* of the Hsp90β–ligands complexes for the last 100 ns of MD simulations, we found Δ*G_bind_* values of −13.73 kcal/mol ± 3.92 kcal/mol for ATP, −10.35 kcal/mol ± 7.15 for GDM, and −6.66 kcal/mol ± 8.47 for RIT (Table 1). The overall trend of binding free energy values followed the series ATP < GDM < RIT.

When comparing the free energy of ATP, GDM, and RIT as derived from experimental dissociation constants (*K_d_*) reported in the literature with triplicates of the theoretical free energy of the same ligands obtained through in silico simulations, we found a correlation of approximately 98.5%. The free energy of RIT is only slightly higher compared to the other ligands (<2 kcal/mol), indicating a good affinity for the N-terminal region of Hsp90β.

## 3. Discussion

Repurposing existing drugs can shorten innovation timelines by 12–17 years, potentially saving $2–3 billion [32,33]. Of course, this approach depends on the availability of specific data such as crystallographic data, which can be difficult to obtain in determined circumstances; as an example, one can cite the difficulty of crystallizing certain proteins or their complexes with a given substrate [34]. However, techniques of artificial intelligence are expected to boost the process of in silico structure modeling [35]. Further progress has been achieved with Cryo-EM, which enables the modeling of large protein structures that was previously impossible to perform [36]. Of course, in silico modeling has its specific costs, such as the necessity of expensive high performance computing equipment [37], but drug libraries for high-throughput screening (HTS) are also very expensive [38]. However, in silico techniques have the merit of democratizing pharmacological research by making it accessible to universities and public research centers [39]. One of the primary advantages of this approach is that the toxicity, efficacy, pharmacokinetic, and pharmacodynamic profiles of these drugs are already established. Successful examples of drug repurposing using computational tools include raloxifene [40], ketoconazole [41], and digoxin [42]. We believe that each technology offers unique advantages based on its specific features, and the optimal approach is to combine them to maximize their benefits [43].

Hsp90β is a complex protein that interacts with dozens of cellular components [44], modulating its functionality. Therefore, it has some interaction regions on its external surface, such as the region that interacts with the co-chaperone CDC37 [29,45]. Trying drugs at these sites would only have specific effects. However, ATP substrates are essential for Hsp90β function. Thus, initial efforts to investigate Hsp90β inhibitors focused on suppressing the ATPase activity of Hsp90β via ligand competition in the ATP active site at the N-terminus. Competitive inhibitors block ATP hydrolysis of Hsp90β and subsequently prevent N-terminal closure of the Hsp90β dimer, which seriously impairs the Hsp90β chaperone cycle essential for client protein maturation.

Investigations of the underlying biological mechanisms of Hsp90 ATPase activity allowed the classification of its inhibitors into (i) those that bind to the N-terminal ATP pocket and (ii) those that affect the C-terminus by preventing dimerization. Natural compounds such as ansamycin, geldanamycin, and radicicol, as well as their derivatives, are highly specific to Hsp90β and competitors of ATP in its active site; however, to the best of our knowledge, they are still toxic or ineffective. For this reason, we chose to reposition the approved drugs in the Hsp90β active sites, which is a type of reverse engineering. To this end, we investigated how the ATP substrate, geldanamycin, and ritonavir inhibitors interacted with the N-terminal site of Hsp90β [18].

The P08238 sequence of Hsp90β belongs to the human interactome, as documented in the IntAct version of 2017 (https://ftp.ebi.ac.uk/pub/databases/intact/current/psimitab/intact-micluster.txt, accessed on 11 January 2018), and is frequently upregulated in many types of solid tumors [46,47]. However, the crystal structure of yeast Hsp90 was obtained by partially deleting the DL region, and showed a closed, compact conformation in the presence of AMP-PNP [adenosine 5′-(β,γ-imido)triphosphate] and Sba1/p23 [48,49]. The fact that the DL region is challenging to map structurally led to the assumption that this region is disordered and flexible, thereby enabling conformational rearrangements of Hsp90β [49,50,51]. This region remains challenging despite the continuous technological progress in resolving protein structures [26]. Therefore, the 5FWK model did not account for the disordered loop that links the N-terminal with the central domain of Hsp90β, but it contained information relative to its ion. Thus, we combined data from PDB and AlphaFold2. The quality scores of the predicted models are highly convergent.

As expected from the PrDos and IUPred servers, several disordered regions were identified along the P08238 sequence, which is consistent with the well-known intrinsic disorder of HSP90β, whose complexes with client proteins are complicated to crystalize [29]. The perfect match of the link loop with the diagnosis as a disordered region by these servers was a type of validation for the threshold to be adopted to detect other similarly disordered regions. The only region whose size was significant was the CD side. Again, this result was expected because it corresponds to the MEEVS motif on the C-terminal tail that allows Hsp90β to establish interactions with several other protein clients [29].

The region of high disorder between amino acids 522 and 583 corresponds to a highly flexible structure that allows Hsp90β to successively close from *close one* to two *close two* stages [29,52]. The disordered regions of the CTD are supposed to enable the two monomers to adapt to conformational changes without breaking their association.

The scoring functions shown in this article have proven to be valuable tools for evaluating model quality, distinguishing good models from wrong models, especially those referring to DL regions. Considering the five models of this report, the ModFOLD8, Molprobity, and QMEAN methods consistently outperformed other quality assessment programs in predicting quality, especially regarding the disordered DL region. The ModFOLD8 and Qmean servers produce accurate estimates of the local and global quality of 3D protein models and have excellent quality prediction scores according to CASP14 [53,54].

The Ramachandran plot was consistent with highly coherent torsion angles, as confirmed by the TM scores. The ERRAT score was associated with low confidence limits of several sequence regions, indicating that the statistical confidence of their folding was lower. However, this may result from a window size that was too restrictive, according to the actual secondary structure being locally scrutinized.

Not considering these disordered regions in RMSD or RMSF analyses confirmed that they were responsible for Hsp90β instability during MD. The structural conformation of disordered regions is complex to predict, given that DL remains a high mobility loop, which contributes to the increase in the mean deviation of the protein as a function of time. However, the RMSD of ATP was independent of these disordered regions and, consequently, not affected by them. Loop mobility is necessary to ensure sufficient protein flexibility for the large conformational changes associated with the functional Hsp90β cycle, which governs its interaction with potential clients [29,44]. Indeed, some reports state that this loop does not influence Hsp90β functionality [55], although evidence shows that every Hsp90β region contributes significantly to its functionality [56]. It has also been proposed that the DL region could act as a highly charged interface for interactions with other proteins, rather than as a connector between domains [57].

H-bonds of ligand interactions with Hsp90β fluctuated in the active site and were rarely permanent during MD simulation of ATP, except for Ser108 and Gly132. GDM was almost as stable as ATP in the active site; however, RIT changed its interactions more frequently over time. However, it remained within the active site during the simulation, demonstrating its potential application in drug repositioning with the benefits of clinical approval.

Cluster analysis is commonly used to determine the most representative protein conformations for protein simulations. The result of any of these analyses is a set of clusters organized hierarchically, with structure populations associated with each cluster and each conformation identified in a specific cluster. In this work, we have observed the interactions made by ATP in the most representative clusters, and we correlated these with the interactions made throughout MD simulations [58], especially regarding the triphosphate region.

The most representative cluster of 3D structures during MD simulation is the statistical notion introduced in this report to simplify the MD interpretation. There is no reason to focus on the interactions between ligand and protein when they do not repeat significantly over time. In all likelihood, a significant phenomenon is likely to occur frequently. This is why consideration of the most relevant cluster makes sense. In addition, it enabled us to compare the chemical interactions of ATP in the native model or of GDM and RIT docked in the same center of mass as ATP with those obtained with the most representative cluster.

Previous studies [59] with crystallized N-terminuses of Hsp90β showed the existence of hydrophobic environments, including the ATP-active side. In this study, we observed a predominance of interactions between Ser108, Val131, Gly132, and Thr179 [59]. These amino acids are described as catalysts for ATP hydrolysis in ADP promoted by Hsp90 [60].

Of the well-known anticancer drugs, GDM was one of the first to inhibit HSP90β, but owing to its high toxicity, it had to be abandoned and only served as the basis for the search for new drugs [18,61]. GDM specifically binds HSP90β competitively to ATP and inhibits HSP90’s ATPase activity [60,62]. However, preclinical trials of GDM have been terminated due to its hepatotoxicity, in vivo instability, and low solubility [63].

RIT demonstrated a higher free energy due to the larger number of rotamers (*n* = 18) compared to ATP and GDM, which promotes greater mobility and adaptation to the binding site. However, the difference of affinity of RIT with Hsp90β compared to the other two ligands is small, and the interactions of RIT at the N-terminal site may promote the inhibition of several Hsp90β functions, leading to cell cycle disruption and apoptosis [21,64].

To date, there is no evidence of inhibition of Hsp90β by RIT at the N-terminus, which motivated us to investigate drug competition at this site. This could lay the groundwork for future research and the design of new drugs based on the chemical interactions identified.

According to its free energy, RIT has a lower affinity for interacting with the active site of Hsp90β than GDM and ATP. The number of rotamers present in RIT (*n* = 18) provides greater mobility within the active site, which favors the search for interactions along the DM and justifies its higher RMSD and lower affinity compared with GDM.

The affinity of RIT may be associated with interactions with the triad Phe129, Gly130, and Val131, as ATP interacts with these amino acids in the same manner. These high-affinity interactions have allowed the identification of critical chemical ligations in the search for anticancer agents through fragment-based approaches and high-performance screening, but these compounds are still under clinical investigation [65]. RIT inhibits MDA-MB-231 xenografts at clinically achievable serum concentrations [66], which suggests that RIT can be transposed into clinical trials at an initial stage for recurrent or metastatic breast cancer. Sustained exposure of MDA-MB-231 cells to daily RIT dosing can deplete Hsp90β [15]. Previous studies have suggested that RIT inhibits Hsp90β by interfering with protein dimerization via attachment to the C-terminal region [67].

## 4. Methodology

### 4.1. Molecular Modeling and Quality Assessment

The 3D model of the heat shock protein Hsp90β, whose sequence was deposited on the UniprotKB server (http://www.uniprot.org, accessed on 20 May 2024) under the accession number P08238 (sequence size: 724 amino acids), was retrieved from the AlphaFold v2.0 (https://alphafold.ebi.ac.uk/entry/P08238, accessed on 20 May 2024) [68] server. The sequence was also used to search for homologous structures in the Protein Data Bank (PDB) [69]. Hsp90β is a homodimer, meaning that chains A and B are identical. Notably, AlphaFold provides structural modeling that includes regions absent in the crystal model (PDB ID: 5WFK), particularly the loops. However, the crystal model presents the homodimer structure with detailed information on ATP binding in the Hsp90β active site. Therefore, we utilized both models to generate structural parameters.

With Modeller v10.2 [70], we created five models based on the monomer data from AlphaFold and information relative to the structure folding as well as ions and substrate positions from the PDB model. We generated five classified models based on the minimum Modeller objective function value for chain A only, which we used for successive analyses. Initially, we aimed to pinpoint disordered regions, as these frequently present challenges for both experimental and theoretical structural studies. Disordered regions are inherently flexible, which makes it exceedingly difficult to model and/or crystallize, and as a result, x-ray diffraction analysis may become unattainable. Therefore, to mitigate any undesirable noise in the models during subsequent quality assessment steps, we eliminated these regions. [71]. Subsequently, we identified the regions of intrinsic disorder using (i) PrDOS [72] and (ii) IUPred2 [73].

The output files of Modeller comprised five models ranked using (i) ModFOLD8 [27], (ii) TM scores [74], (iii) Ramachandran plots and all-atom structure validation for macromolecular crystallography with Molprobity [75,76] through the PSVs 1.5 meta server, (iv) QMEAN [28], and (v) Errat [77].

The final refinement of the best 3D model of Hsp90β was performed using the ModRefine server [78] in addition to analyzing the electrostatic potential using the Adaptive Poisson–Boltzmann Solver (APBS) in APBS–PDB2PQR (version 3.6.1) suite web server (https://server.poissonboltzmann.org/pdb2pqr, accessed on 20 May 2024) [79] at pH 7.4. PDB2PQR automates the preparation of structures for subsequent analysis by adding atomic charges and radius parameters to PDB models and estimating the titration states and protonation [80]. APBS solves the equations of continuum electrostatics for any biomolecular structure by calculating their electrostatic and solvation properties using the implicit solvent model of Poisson–Boltzmann (PB), which provides a global solution suited to visualization and structural analyses. Hydrophobicity analysis of the active sites was performed using the Kyte and Doolittle scale [81] implemented in Chimera [82].

### 4.2. Ligand Parameterization and Molecular Docking

**Figure 7:**
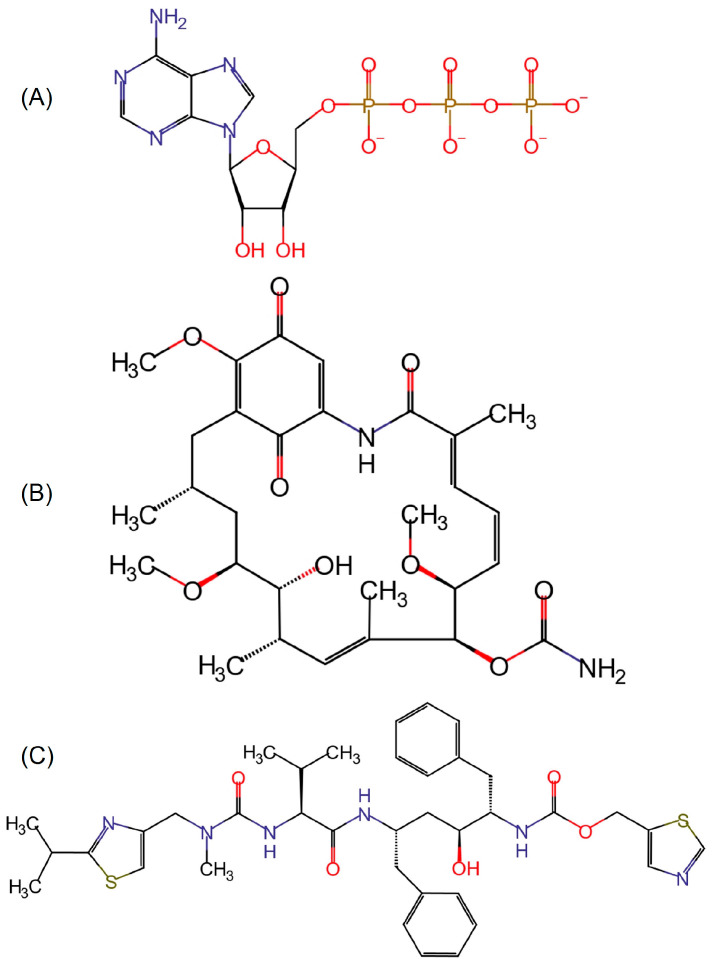
Hsp90β ligands. (**A**): ATP, (**B**): geldanamycin, and (**C**): ritonavir.

The 3D structure of ATP was retrieved from the PDB model 5WFK, while those of GDM and RIT were obtained from PubChem (https://pubchem.ncbi.nlm.nih.gov/, accessed on 20 May 2024) with accessions 5,288,382 and 392,622, respectively. The 2D structures were drawn using MarvinSketch 15.3.26 (ChemAxon Ltd., http://www.chemaxon.com/, Budapest, Hungary) (Figure 7). Subsequently, the restrained electrostatic potential (RESP) method [83], provided by the Ambertools package [84], was used to fit the atomic partial charges of the optimized structures. Finally, the Amber99SB force field [85] and the general AMBER force field (GAFF) [86] were used to define bonds, angles, and torsions.

Molecular docking calculations were conducted with DOCK 6.9 [87], utilizing input structures generated through Chimera 1.14 [82]. The determination of the binding site of Hsp90β was facilitated by identifying the center of mass of the substrate (ATP), which corresponds to the most representative cluster of Hsp90β, calculated employing the Gromos method [88], This center of mass served as a reference point for defining the center of the docking box for the molecular docking simulations of GDM and RIT.

The DOCK accessory program sphgen [89] was used to precompute the energy interactions between a dummy probe atom and all receptor atoms on a 0.3 Å resolution grid within the box. For these experiments, we used (i) the Lennard–Jones potential with 6–9 attractive and repulsive exponents for modeling interactions, and (ii) the Coulomb potential with a distance-dependent dielectric coefficient of ε = 4r to model the electrostatic interactions. This resulting grid formed the basis for computing the single grid energy (SGE) score used in all tests in this study [87]. Each scan produced ten conformations for each ligand, and those with the best scores were used for molecular dynamics (MD) simulations.

### 4.3. Interaction Modeling

We mapped the interactions between Hsp90β and ATP, GDM, and RIT using DiscoveryStudio v.2021 (2D representation) [90] and LigandScout 4.4.9 [91] (3D representation). LigandScout was configured to generate models of chemical bonds based on the functional groups of the amino acids that make up the active site, and to align the models to ensure their overlapping. Once the interactions of all possible conformer inferences of both the amino acids of the active site and the ligands considered were identified, LigandScout superimposed them to generate a final model. Then, we analyzed, with DiscoveryStudio v.2021 [90], the most representative Hsp90β–ligand clusters obtained by MD to determine whether similar interactions were engaged with the amino acids constituting the binding site.

### 4.4. Molecular Dynamics

It is well established that the docking poses of ligands are not reliable for analyzing their chemical interactions within protein complexes. To address this issue, we used the docking poses of the ligands as starting points for MD simulations. We considered only the chemical interactions from the most representative structures of the RIT and GDM complexes with Hsp90β, identified among three MD replicates, as reliable.

MD simulation were performed using Gromacs 2023.2 [92], with the force field AMBER99SB [93] at pH 7.4, with deprotonated Glu and Asp and protonated Arg and Lys for all proteins. The electrostatic interactions were calculated using the particle mesh Ewald (PME) method with a cutoff of 1.2 nm [94]. The same cutoff value of 1.2 nm was used for the van der Waals interactions. To treat long-range Coulomb interactions, Lennard–Jones interactions were cut off at 1.0 nm with analytical tail corrections for the long-range dispersion.

Hsp90β was centered in a cubic box of TIP3P water molecules [95], the box was extended by 1.2 nm, and the appropriate numbers of Na^+^ and Cl^−^ counterions were added to ensure system neutralization. The setup was then energy-minimized using the steepest descent algorithm by running 25,000 steps to optimize the complex structure. Each system of Hsp90β and its ligands (ATP, GDM, or RIT) was then simulated by heating it in the NVT ensemble using the V-rescale thermostat [96] for 5 ns from 0 to 310 K, equilibrated in an NPT ensemble of 1.0 bar using the Parrinello–Rahman barostat [97] for 5 ns. The LINCS algorithm was used to constrain all the bonds. The restraining forces of the protein backbone, Mg^2+^, substrate, and ligands were set to 1 kcal/mol, whereas the solvent and counter ions were allowed to move during the heating, equilibration, and production phases. During the production run, position restraints were entirely removed.

Finally, the systems were simulated in triplicate, with random seed for 200 ns each, under periodic boundary conditions at 310 K temperature and 1.0 bar pressure. Root mean square deviation (RMSD), root mean square fluctuation (RMSF), and clustering analyses were performed using the gmx cluster with the GROMOS method [88,98]. Clustering analysis was performed with a cutoff of 4.0 Å. The hydrogen bond occupation was calculated with CPPTRAJ using a geometric criterion. The H-bond was calculated using a cutoff distance and angle between the active site of Hsp90β and the ligands ATP, GDM, and RIT of 3.5 Å and 120°, respectively.

### 4.5. Free Energy Calculation Using MMPBSA

One of the most often used techniques for calculating binding free energy is molecular mechanics/Poisson–Boltzmann (generalized Born) surface area—MM/PB(GB)SA. It has been demonstrated to balance precision with computational efficiency, particularly when working with large systems [99].

Here, free energy calculations were performed with gmx_MMPBSA using Gromacs trajectory and topology files. The last 100 ns of the MD simulation were used to calculate the binding free energy of the complexes. The binding free energy (Δ*G_binding_*) of each ligand complexed with Hsp90β was calculated using Formula (1).

(1) ∆*G_binding_* = ∆*H* − *T*∆*S*

In Formula (1), Δ*H* corresponds to the binding enthalpy and represents the conformational entropy after ligand binding. When the entropic term is omitted, the computed value is the effective free energy, which is typically sufficient for comparing the relative binding free energy of related ligands [100,101]. To estimate a theoretical value of *K_i_* or *IC*_50_, we correlated the free energy values derived from experimental constants with the free energy values obtained from simulations (see Formulas (2)–(4)).

(2) $${\Delta G}_{bind,exp0}=-RT\ln\left(\frac{1}{Ki}\right)$$

(3) $${\Delta G}_{bind,exp0}=-RT\ln{IC}_{50}$$

(4) $${\Delta G}_{bind,exp0}=RTln\frac{K_{d}}{C_{0}}$$

where *C*_0_ is the concentration that defines the standard state, conventionally set to 1 mol/L, R is the gas constant (8.3144 J K^−1^mol^−1^, equivalent to 1.9872 cal K^−1^mol^−1^), T is the absolute temperature (Kelvin), and Δ*G_bind_* is the binding free energy, which in the case of *K_d_* refers to the energy after the complex dissociation. An index file was generated at the start of the MD simulation and the output was saved in DAT (.dat) and CSV (.csv) formats. The results were analyzed with the *ana* module of the gmx_MMBPSA. This approach of correlating inhibition constants with binding free energy has been employed in a previous study [31].

## 5. Conclusions

In this report, we have exemplified how computational methods can aid in drug repositioning and accelerate the development of new treatments and therapies by generating new hypotheses, thereby potentially shortening the pharmaceutical development process.

We analyzed the interaction of ATP, GDM, and RIT with Hsp90β to better understand the possible interactions between GDM and RIT inhibition of HSP90s.

It should be noted that the DL region linking the N-terminus and CD is disordered and promotes increased mobility of the Hsp90β structure. However, when not considered, the DL region had no direct influence on the interactions between Hsp90β and ATP, GDM, or RIT. Its omission from the 3D structure does not cause any disadvantage to the model or to the information obtained on the interactions between potential drugs and their active sites.

The chemical environment of the active site of Hsp90β is predominantly hydrophobic but contains amino acid hydrogen donors and acceptors. Amino acid donors and hydrogen receptors interact predominantly with the ATP triphosphate and RIT thiazole regions identified via molecular docking and dynamics.

The MMPBSA method proved effective in ranking ATP, GDM, and RIT by demonstrating a strong correlation between the free energy derived from molecular dynamics calculations and those based on the dissociation constants reported in the literature. Consequently, MMPBSA’s contribution could expedite the repositioning of other potential drugs and even the development of new drugs targeting Hsp90β. Combining the information on interactions between ATP and RIT as well as free energy could open the possibility of designing an inhibitor with greater affinity and action for the interaction at the N-terminus of Hsp90β, increasing the chances of designing new drugs to inhibit Hsp90β. The approach presented in this study intends to extend drug repositioning to other target proteins to increase the spectrum of cancer chemotherapies.

## Figures and Tables

**Table 1:** Estimated free energy (kcal/mol) of ligands.

Ligands	ΔH, Average	−TΔS, Average	ΔG	Experimental
ATP	−30.43 ± 2.30	16.69 ± 0.12	−13.73 ± 3.92	−9.07
GDM	−20.98 ± 2.90	10.62 ± 3.39	−10.35 ± 7.15	−8.39
RIT	−16.81 ± 2.62	10.16 ± 1.83	−6.66 ± 8.47	−7.24

## Data Availability

All the data produced by this research are contained in this report and the Supplementary Materials.

## Supplementary Materials

The following supporting information can be downloaded at: https://www.mdpi.com/article/10.3390/ijms25168782/s1.
