# Supplementary material for: Structural Characterization of Heat Shock Protein 90β and Molecular Interactions with Geldanamycin and Ritonavir: A Computational Study
Source: Int J Mol Sci. 2024 Aug 12;25(16):8782. doi: 10.3390/ijms25168782 (PMC11354266; doi:10.3390/ijms25168782)
Supplement: Supplementary file 1 [file ijms-25-08782-s001.zip › LimaEtAl_SM/FigS1.docx]

**
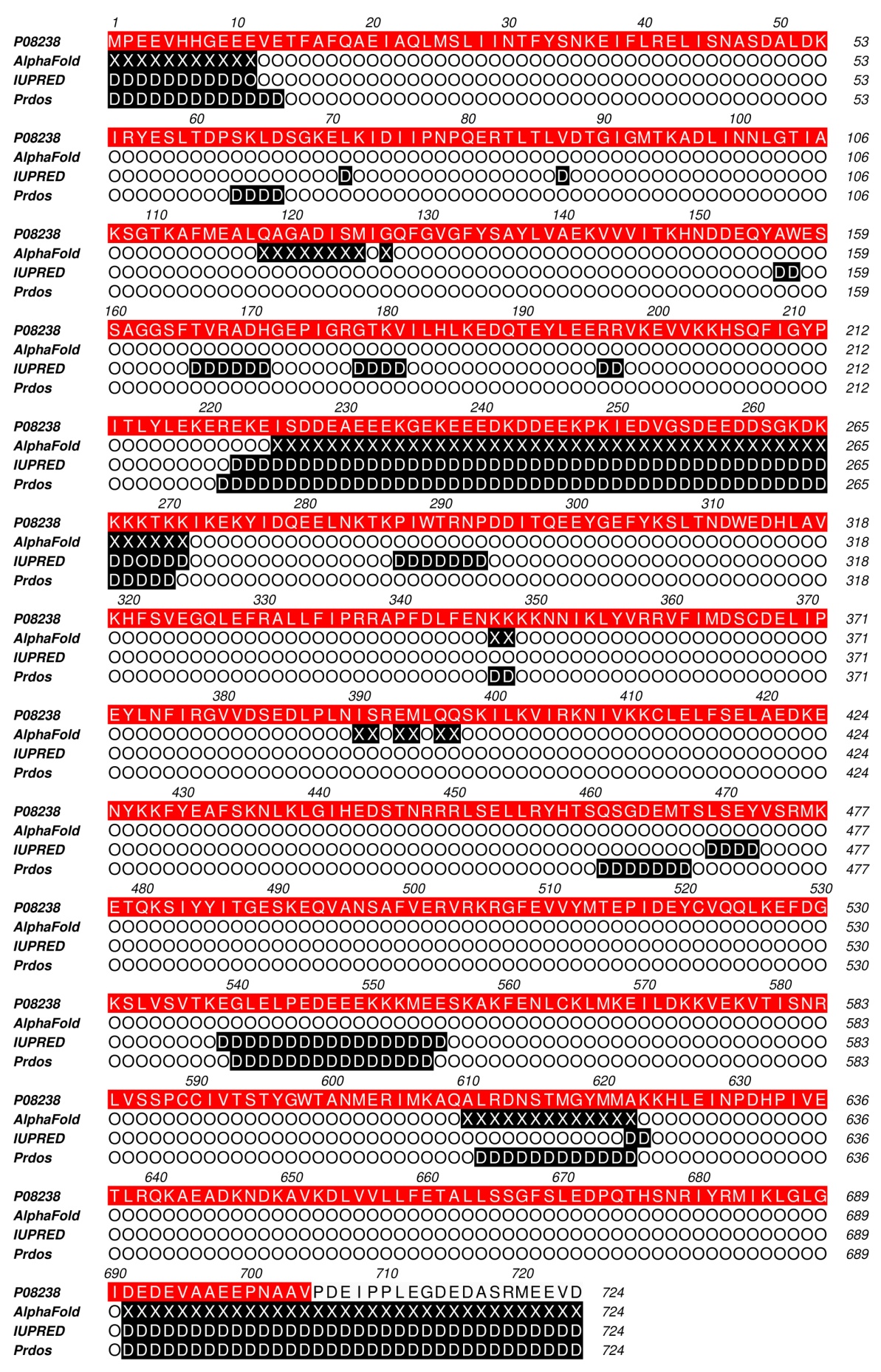
**

**Figure S1**. 1D alignment of the low-confidence regions predicted by AlphaFol2 and the regions of intrinsic disorder predicted by PrDos and IUPred for Hsp90β. Notably, the NTD (10 AA), DL (46 AA), and CTD (33 AA) regions exhibited disorder levels exceeding the 50% threshold (see Figure 2).
