# Supplementary material for: Structural Characterization of Heat Shock Protein 90β and Molecular Interactions with Geldanamycin and Ritonavir: A Computational Study
Source: Int J Mol Sci. 2024 Aug 12;25(16):8782. doi: 10.3390/ijms25168782 (PMC11354266; doi:10.3390/ijms25168782)
Supplement: Supplementary file 1 [file ijms-25-08782-s001.zip › LimaEtAl_SM/FigS8.docx]

**
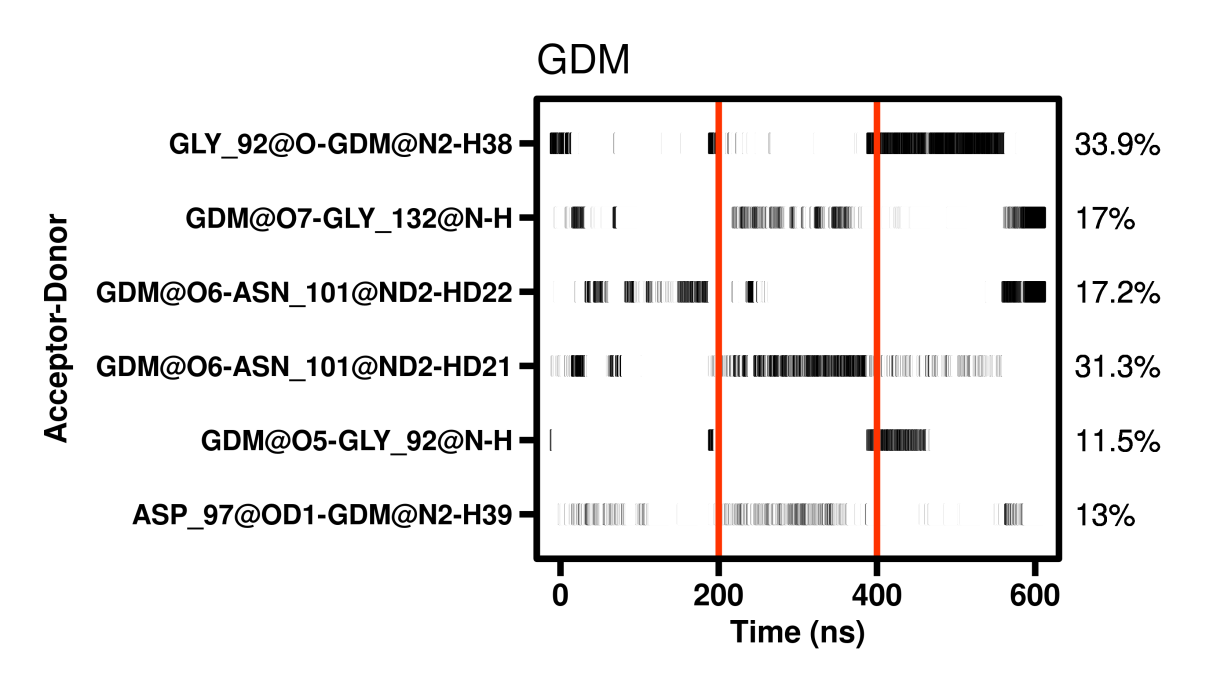
**

**Figure S8**. Hydrogen bonds identified by MD concatenate the trajectories of Hsp90β-GDM complex as a function of time.
