# Supplementary material for: Structural Characterization of Heat Shock Protein 90β and Molecular Interactions with Geldanamycin and Ritonavir: A Computational Study
Source: Int J Mol Sci. 2024 Aug 12;25(16):8782. doi: 10.3390/ijms25168782 (PMC11354266; doi:10.3390/ijms25168782)
Supplement: Supplementary file 1 [file ijms-25-08782-s001.zip › LimaEtAl_SM/Tabelas_S1-7.docx]

|  | TM-Score | | | | Molprobity (Ramachandran Plot) % | QMEAN | | ModFOLD 8 | | Errat |
| --- | --- | --- | --- | --- | --- | --- | --- | --- | --- | --- |
|  | Alphafold | PDB | Alphafold  (RMSD) | PDB  (RMSD) |  | Score | Z-score | Confidence and  P-value | Global model quality score |  |
| Model 1 | 0.9811 | 0.9789 | 0.136 | 1.467 | 95.80 | 0.71 | -1.89 | 7.84E-05 | 0.576 | 72.15 |
| Model 2 | 0.9651 | 0.9708 | 2.189 | 1.858 | 95.80 | 0.71 | -1.97 | 7.88E-05 | 0.576 | 72.52 |
| Model 3 | 0.9736 | 0.9825 | 1.629 | 1.306 | 95.60 | 0.70 | -2.42 | 7.91E-05 | 0.576 | 73.53 |
| Model 4 | 0.9605 | 0.9821 | 2.133 | 1.439 | 95.50 | 0.71 | -1.70 | 8.20E-05 | 0.574 | 76.14 |
| Model 5 | 0.9748 | 0.9836 | 1.548 | 1.285 | 95.60 | 0.71 | -1.79 | 8.78E-05 | 0.571 | 73.13 |
| Average | 0.971 | 0.980 | 1.527 | 1.471 | 95.66 | 0.708 | -1.954 | 8.12E-05 | 0.574 | 73.49 |
| Std deviation | 0.008 | 0.005 | 0.829 | 0.231 | 0.134 | 0.004 | 0.280 | 3.93E-06 | 0.002 | 1.57 |
| Sample | 5 | 5 | 5 | 5 | 5 | 5 | 5 | 5 | 5 | 5 |
| Std error | 0.004 | 0.002 | 0.371 | 0.103 | 0.060 | 0.002 | 0.125 | 1.76E-06 | 9.58E-04 | 0.70 |

**Table S1.** Evaluation of the quality of the five HSP90β models og this study.

**Table S2.** Interactions of ATP with Hsp90β before MD initiation.

| Initial Model | | |
| --- | --- | --- |
| Bond's type | AA | Distance (Å) |
| H-bond | Asn46, Asp88, Ser108, Gly109, Thr110, Phe129, Val131, Gly132, | 2.08, 2.72, 2.12, 2.65, 2.56, 2.89, 2.70, 2.13, |
| Carbon Hydrogen bond | Lys107, Gly130 | 3.04, 2.86, |
| Pi-Alkul | Ala50, Met93 | 4.69, 5.07 |
| Amide-Pi Stacked | Asn46/Ala47 | 5.06 |
| Attractive Charge | Arg392, Mg^2+^ | 4.62, 5.21 |
| metal Acceptor | Mg^2+^ | 2.77 |

**Table S3**. Interactions of the best cluster of concatenated MD trajectories of ATP with Hsp90β.

| Best Cluster | | |
| --- | --- | --- |
| Bond's type | AA | Distance (Å) |
| H-bond | Asn46, Asp88, Ser108, Gly109, Thr110, Gly130, Val131, Gly132, Pge133, Thr179 | 1.90, 2.25, 1.50, 1.75, 1.81, 3.03, 2.07, 1.96, 2.24, 2.30 |
| Carbon Hydrogen bond | Gly127 | 2.68 |
| Pi-Alkul | Ala50, Met93 | 4.39, 5.11 |
| Pi-Sulfur | Met93 | 4.45 |
| Attractive Charge | Arg392, Mg^2+^ | 5.47, 5.15 |
| metal Acceptor | Mg^2+^ | 1.93 |

**Table S4**. Interactions of geldanamycin (GDM) after docking with Hsp90β.

| Initial Model GDM | | |
| --- | --- | --- |
| Bond's type | AA | Distance (Å) |
| H-bond | Asn46, Lys53, Gly92, Phe133 | 2.28, 2.95, 1.96, 2.41 |
| Carbon Hydrogen bond | Asp88, Thr104, Gly130 | 2.62, 2.86, 2.62 |
| Alkyl | Ala47, Ala50, Lys53, Lys107 | 3.94, 4.93, 3.50, 3.81 |

**Table S5.** Interactions of ritonavir (RIT) after docking in Hsp90β.

| Initial Model RIT | | |
| --- | --- | --- |
| Bond's type | AA | Distance (Å) |
| H-bond | Asn101, Phe129, Gly130, Val131, Phe133 | 2.08, 2.49, 2.56, 2.68, 2.64 |
| Carbon Hydrogen bond | Asn46, Ser108 | 2.82, 2.96 |
| Alkyl | Arg392 | 5.21 |
| Pi-Alkyl | Leu43, Ala47, Ala50, Met93, Phe129,Val131, Val181 | 5.30, 4.05, 4.01, 4.32, 5.40, 5.38, 5.35 |
| Pi-Anion | Asp49 | 4.36 |
| Pi-Sulfur | Met125, Phe133 | 5.29, 5.77 |
| Pi-Pi T-Shared | Phe133 | 5.55 |

**Table S6.** Interactions of geldanamycin (GDM) with the best Hsp90β cluster.

| Best cluster Hsp90β-GDM | | |
| --- | --- | --- |
| Bond's type | AA | Distance (Å) |
| H-bond | Met93, Asn101, Met125 and Gly132 | 2.77, 2.67/2.11, 2.84, 2.58 |
| Carbon Hydrogen bond | Lys53, Ser108, Gly127, Gly130 and Gly132 | 2.39, 2.69, 2.88, 2.49, 2.47 |
| Alkyl | Ala50, Lys53, Ile91, Met93, Met125 and Val181 | 4.02, 4.32, 4.88, 5.25, 5.02, 5.25 |
| Pi-Alkyl | Phe133 | 4.59 |

**Table S7.** Interactions made by ritonavir (RIT) with the best cluster of Hsp90β.

| Best cluster Hsp90β-RIT | | |
| --- | --- | --- |
| Bond's type | AA | Distance (Å) |
| H-bond | Asn101, Gly130, Val131 and Gly132 | 2.15, 2.94, 3.10, 2.35 |
| Carbon Hydrogen bond | Asn46, Asn101, Gly127 and Gly130 | 2.71, 2,62, 2.53, 2.85 |
| Pi-Alkyl | Leu43, Ala47, Ala50, Met93, Met125 and Phe133 | 5.39, 4.83, 4.25 / 5.50, 5.03, 4.67 |
| Amide-Pi | Asp49 | 4.98, |
| Pi-Pi | Phe113 | 5.69 |
| Pi-Sigma | Lys53 | 2.73 |
