# Supplementary material for: Structural Characterization of Heat Shock Protein 90β and Molecular Interactions with Geldanamycin and Ritonavir: A Computational Study
Source: Int J Mol Sci. 2024 Aug 12;25(16):8782. doi: 10.3390/ijms25168782 (PMC11354266; doi:10.3390/ijms25168782)
Supplement: Supplementary file 1 [file ijms-25-08782-s001.zip › Tables S1-7.pdf]

Table S1. Evaluation of the quality of the five HSP90β models og this study.

	TM-Score					QMEAN		ModFOLD 8		
	Alphafold	PDB	Alphafold (RMSD)	PDB (RMSD)	Molprobity (Ramachandran Plot) %	Score	Z-score	Confidence and P-value	Global model quality score	Errat
Model 1	0.9811	0.9789	0.136	1.467	95.80	0.71	-1.89	7.84E-05	0.576	72.15
Model 2	0.9651	0.9708	2.189	1.858	95.80	0.71	-1.97	7.88E-05	0.576	72.52
Model 3	0.9736	0.9825	1.629	1.306	95.60	0.70	-2.42	7.91E-05	0.576	73.53
Model 4	0.9605	0.9821	2.133	1.439	95.50	0.71	-1.70	8.20E-05	0.574	76.14
Model 5	0.9748	0.9836	1.548	1.285	95.60	0.71	-1.79	8.78E-05	0.571	73.13
Average	0.971	0.980	1.527	1.471	95.66	0.708	-1.954	8.12E-05	0.574	73.49
Std deviation	0.008	0.005	0.829	0.231	0.134	0.004	0.280	3.93E-06	0.002	1.57
Sample	5	5	5	5	5	5	5	5	5	5
Std error	0.004	0.002	0.371	0.103	0.060	0.002	0.125	1.76E-06	9.58E-04	0.70

Table S2. Interactions of ATP with Hsp90β before MD initiation.

Initial Model		
Bond's type	AA	Distance (Å)
H-bond	Asn46, Asp88, Ser108, Gly109, Thr110, Phe129, Val131, Gly132,	2.08, 2.72, 2.12, 2.65, 2.56, 2.89, 2.70, 2.13,
Carbon Hydrogen bond	Lys107, Gly130	3.04, 2.86,
Pi-Alkul	Ala50, Met93	4.69, 5.07
Amide-Pi Stacked	Asn46/Ala47	5.06
Attractive Charge	Arg392, Mg^{2+}	4.62, 5.21
metal Acceptor	Mg^{2+}	2.77

Table S3. Interactions of the best cluster of concatenated MD trajectories of ATP with Hsp90β.

Best Cluster		
Bond's type	AA	Distance (Å)
H-bond	Asn46, Asp88, Ser108, Gly109, Thr110, Gly130, Val131, Gly132, Pge133, Thr179	1.90, 2.25, 1.50, 1.75, 1.81, 3.03, 2.07, 1.96, 2.24, 2.30
Carbon Hydrogen bond	Gly127	2.68
Pi-Alkul	Ala50, Met93	4.39, 5.11
Pi-Sulfur	Met93	4.45
Attractive Charge	Arg392, Mg^{2+}	5.47, 5.15
metal Acceptor	Mg^{2+}	1.93

Table S4. Interactions of geldanamycin (GDM) after docking with Hsp90β.

Initial Model GDM		
Bond's type	AA	Distance (Å)
H-bond	Asn46, Lys53, Gly92, Phe133	2.28, 2.95, 1.96, 2.41
Carbon Hydrogen bond	Asp88, Thr104, Gly130	2.62, 2.86, 2.62
Alkyl	Ala47, Ala50, Lys53, Lys107	3.94, 4.93, 3.50, 3.81

Table S5. Interactions of ritonavir (RIT) after docking in Hsp90β.

Initial Model RIT		
Bond's type	AA	Distance (Å)
H-bond	Asn101, Phe129, Gly130, Val131, Phe133	2.08, 2.49, 2.56, 2.68, 2.64
Carbon Hydrogen bond	Asn46, Ser108	2.82, 2.96
Alkyl	Arg392	5.21
Pi-Alkyl	Leu43, Ala47, Ala50, Met93, Phe129,Val131, Val181	5.30, 4.05, 4.01, 4.32, 5.40, 5.38, 5.35
Pi-Anion	Asp49	4.36
Pi-Sulfur	Met125, Phe133	5.29, 5.77
Pi-Pi T-Shared	Phe133	5.55

Table S6. Interactions of geldanamycin (GDM) with the best Hsp90β cluster.

Best cluster Hsp90β-GDM		
Bond's type	AA	Distance (Å)
H-bond	Met93, Asn101, Met125 and Gly132	2.77, 2.67/2.11, 2.84, 2.58
Carbon Hydrogen bond	Lys53, Ser108, Gly127, Gly130 and Gly132	2.39, 2.69, 2.88, 2.49, 2.47
Alkyl	Ala50, Lys53, Ile91, Met93, Met125 and Val181	4.02, 4.32, 4.88, 5.25, 5.02, 5.25
Pi-Alkyl	Phe133	4.59

Table S7. Interactions made by ritonavir (RIT) with the best cluster of Hsp90β.

Best cluster Hsp90β-RIT		
Bond's type	AA	Distance (Å)
H-bond	Asn101, Gly130, Val131 and Gly132	2.15, 2.94, 3.10, 2.35
Carbon Hydrogen bond	Asn46, Asn101, Gly127 and Gly130	2.71, 2,62, 2.53, 2.85
Pi-Alkyl	Leu43, Ala47, Ala50, Met93, Met125 and Phe133	5.39, 4.83, 4.25 / 5.50, 5.03, 4.67
Amide-Pi	Asp49	4.98,
Pi-Pi	Phe113	5.69
Pi-Sigma	Lys53	2.73
