# Supplementary material for: Structural Characterization of Heat Shock Protein 90β and Molecular Interactions with Geldanamycin and Ritonavir: A Computational Study
Source: Int J Mol Sci. 2024 Aug 12;25(16):8782. doi: 10.3390/ijms25168782 (PMC11354266; doi:10.3390/ijms25168782)
Supplement: Supplementary file 1 [file ijms-25-08782-s001.zip › Figure S2.pdf]

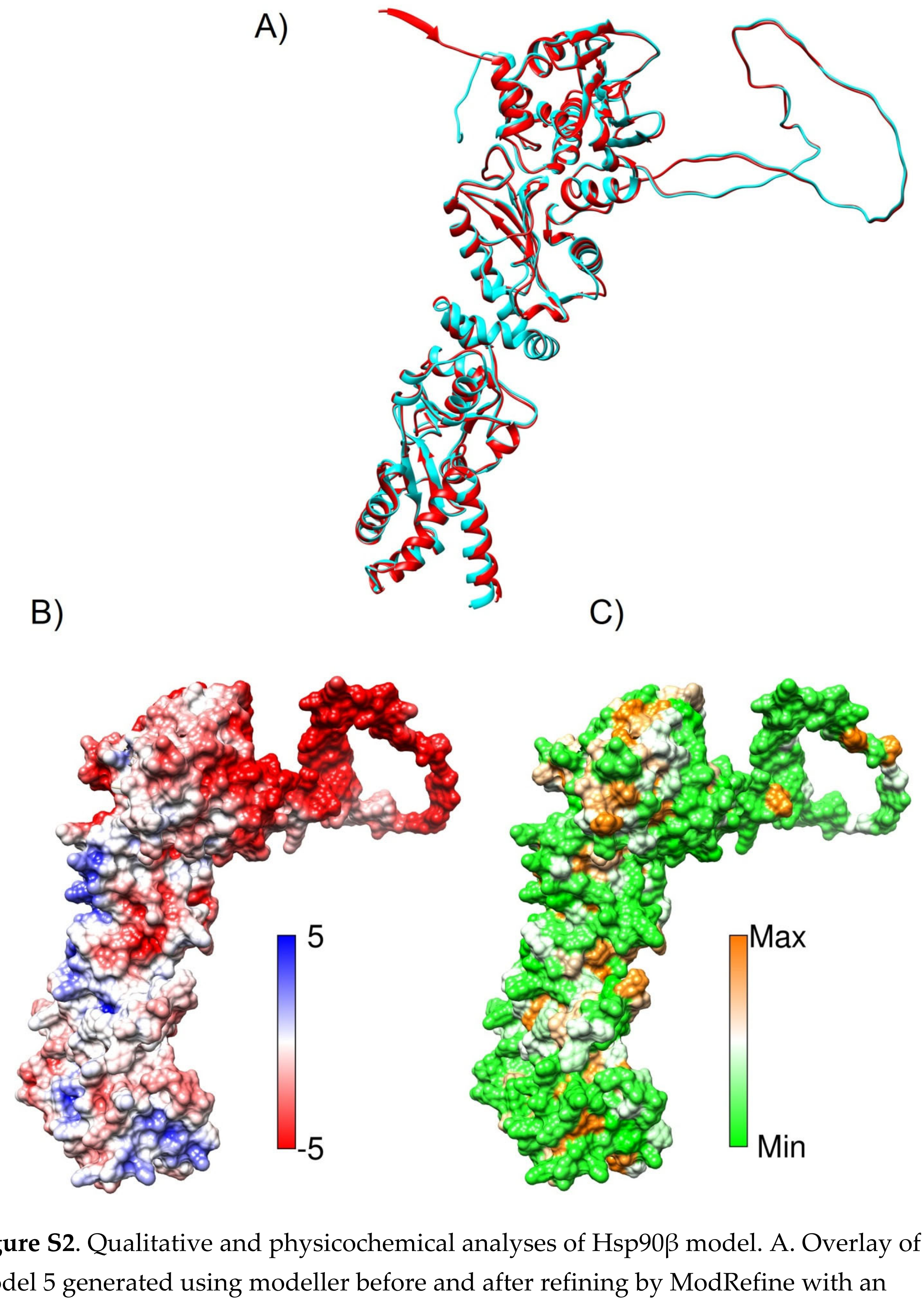

Figure S2. Qualitative and physicochemical analyses of Hsp90β model. A. Overlay of model 5 generated using modeller before and after refining by ModRefine with an RMSD of 2.3Å; B. Electrostatic potential (Kb.T.ec−1) – red: negative, blue: positive; C. Hydropathy: hydrophobic (orange) and hydrophilic (green) residues.
