# Supplementary material for: Structural Characterization of Heat Shock Protein 90β and Molecular Interactions with Geldanamycin and Ritonavir: A Computational Study
Source: Int J Mol Sci. 2024 Aug 12;25(16):8782. doi: 10.3390/ijms25168782 (PMC11354266; doi:10.3390/ijms25168782)
Supplement: Supplementary file 1 [file ijms-25-08782-s001.zip › Figure S3.pdf]

(A)

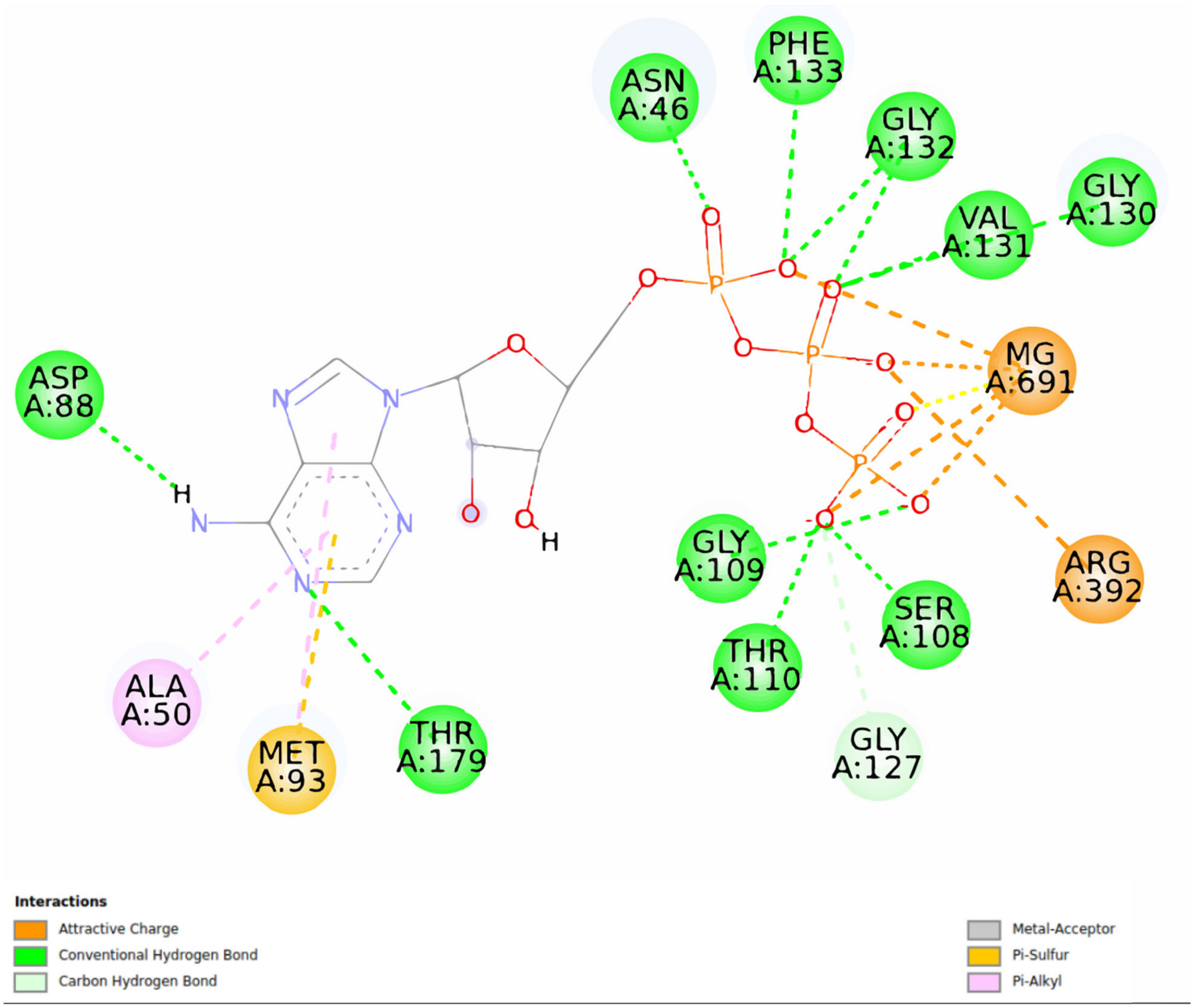

ASN A:46
PHE A:133
GLY A:132
GLY A:130
VAL A:131
MG A:691
ASP A:88
ARG A:392
GLY A:109
SER A:108
THR A:110
GLY A:127
ALA A:50
MET A:93
THR A:179
Interactions
Attractive Charge
Conventional Hydrogen Bond
Carbon Hydrogen Bond
Metal-Acceptor
Pi-Sulfur
Pi-Alkyl

(B)

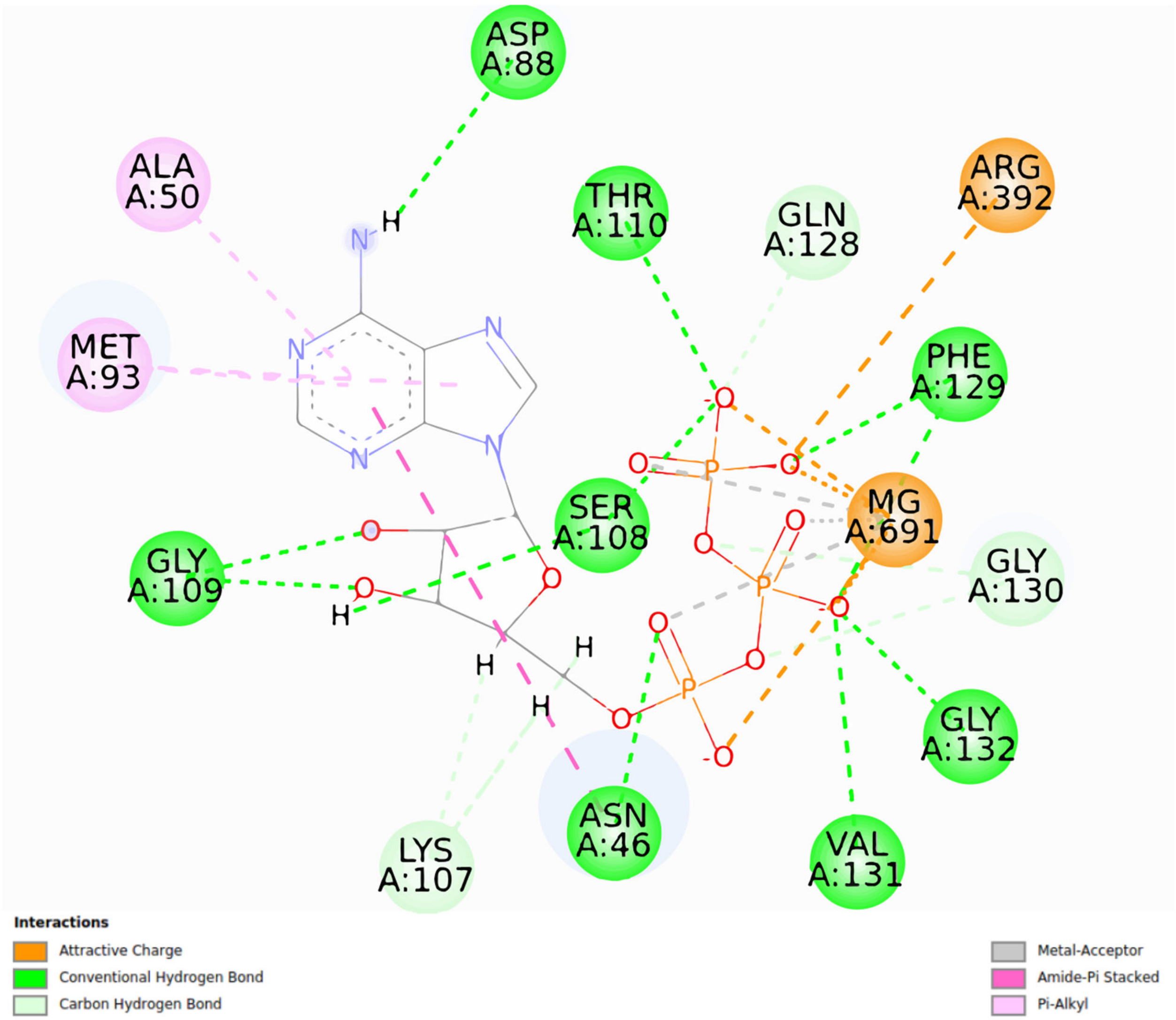

Figure S3. 2D interaction map of ATP complexed with HSP90β before simulation (A) and in the best cluster of concatenated DM trajectories (B).
