# Supplementary material for: Structural Characterization of Heat Shock Protein 90β and Molecular Interactions with Geldanamycin and Ritonavir: A Computational Study
Source: Int J Mol Sci. 2024 Aug 12;25(16):8782. doi: 10.3390/ijms25168782 (PMC11354266; doi:10.3390/ijms25168782)
Supplement: Supplementary file 1 [file ijms-25-08782-s001.zip › Figure S4.pdf]

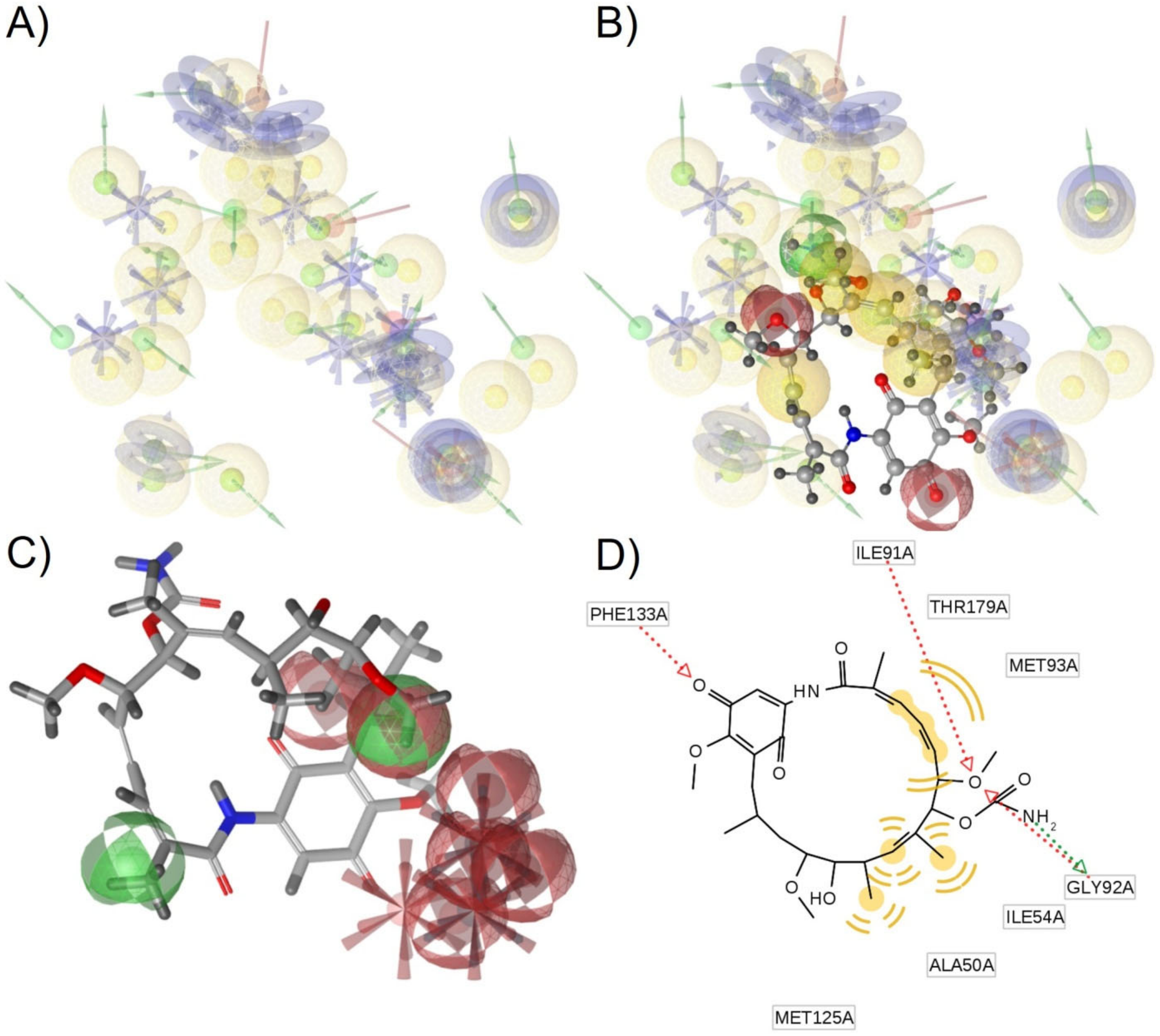

Figure S4. 3D interaction analysis of GDM docked in the N-terminal active site 615 of the best cluster of Hsp90β. A. Map of the functional groups of Hsp90β. B. Interaction between GDM and Hsp90β. C. Interaction foci of GDM with Hsp90β: H-bond acceptors (red) and H-bond donors (green). D. 2D representation of GDM interactions. Yellow spheres represent hydrophobic interactions, blue spheres represent aromatic rings, red arrows indicate H-bond acceptors, and green arrows indicate H-bond donors.
