# Supplementary material for: Structural Characterization of Heat Shock Protein 90β and Molecular Interactions with Geldanamycin and Ritonavir: A Computational Study
Source: Int J Mol Sci. 2024 Aug 12;25(16):8782. doi: 10.3390/ijms25168782 (PMC11354266; doi:10.3390/ijms25168782)
Supplement: Supplementary file 1 [file ijms-25-08782-s001.zip › Figure S5.pdf]

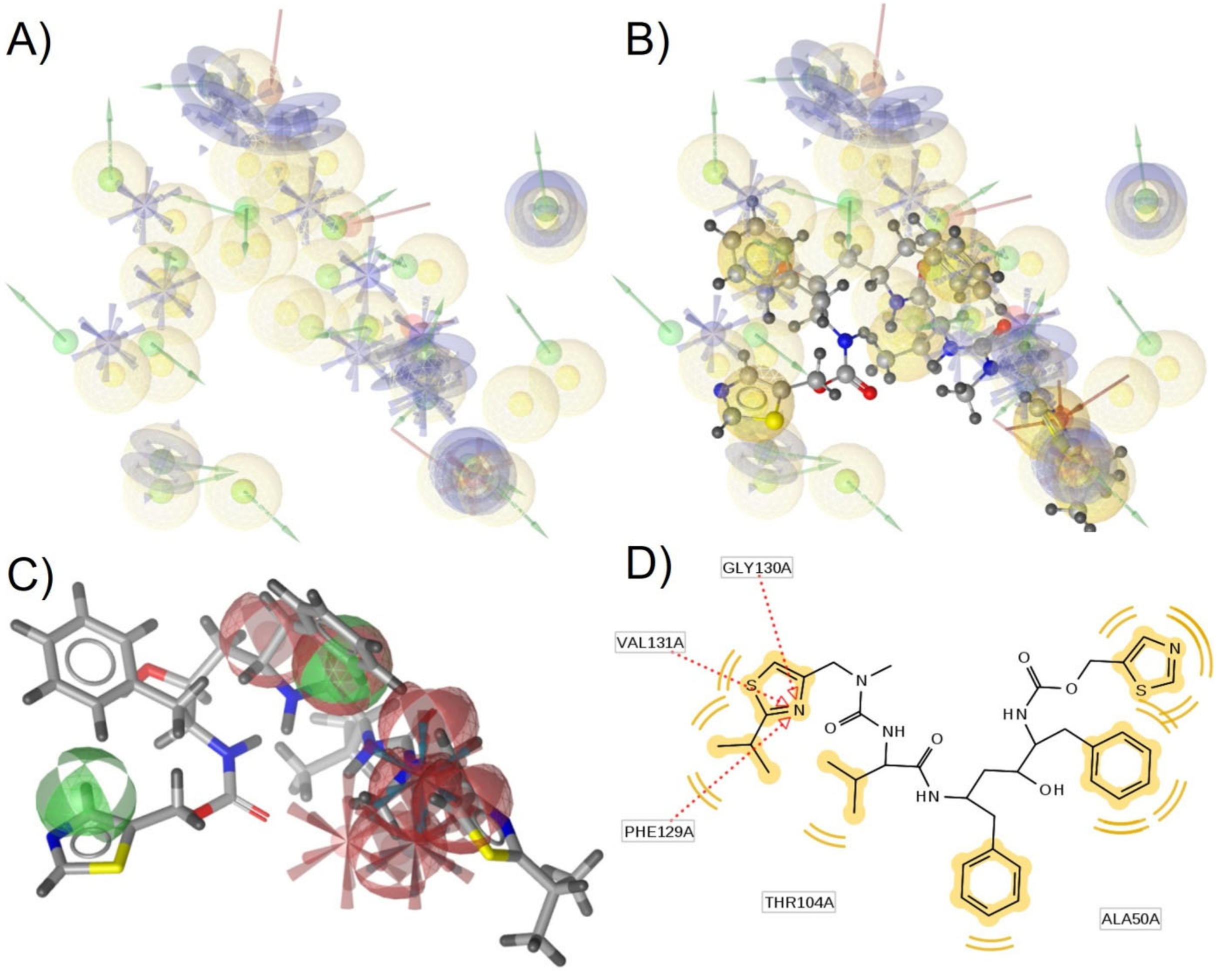

Figure S5. 3D interaction analysis of RIT docked in the N-terminal active site of the best Hsp90β cluster. A. Map of the functional groups of Hsp90β. B. Interaction of RIT with Hsp90β. C. Interaction foci of RIT with Hsp90β: H-bond acceptors (red) and H-bond donors (green). D. 2D representation of RIT interactions. Yellow spheres represent hydrophobic interactions; blue spheres represent aromatic rings; red arrows indicate H-bond acceptors; and green arrows depict the presence of H-bond donors.
