# Supplementary material for: Structural Characterization of Heat Shock Protein 90β and Molecular Interactions with Geldanamycin and Ritonavir: A Computational Study
Source: Int J Mol Sci. 2024 Aug 12;25(16):8782. doi: 10.3390/ijms25168782 (PMC11354266; doi:10.3390/ijms25168782)
Supplement: Supplementary file 1 [file ijms-25-08782-s001.zip › Figure S7.pdf]

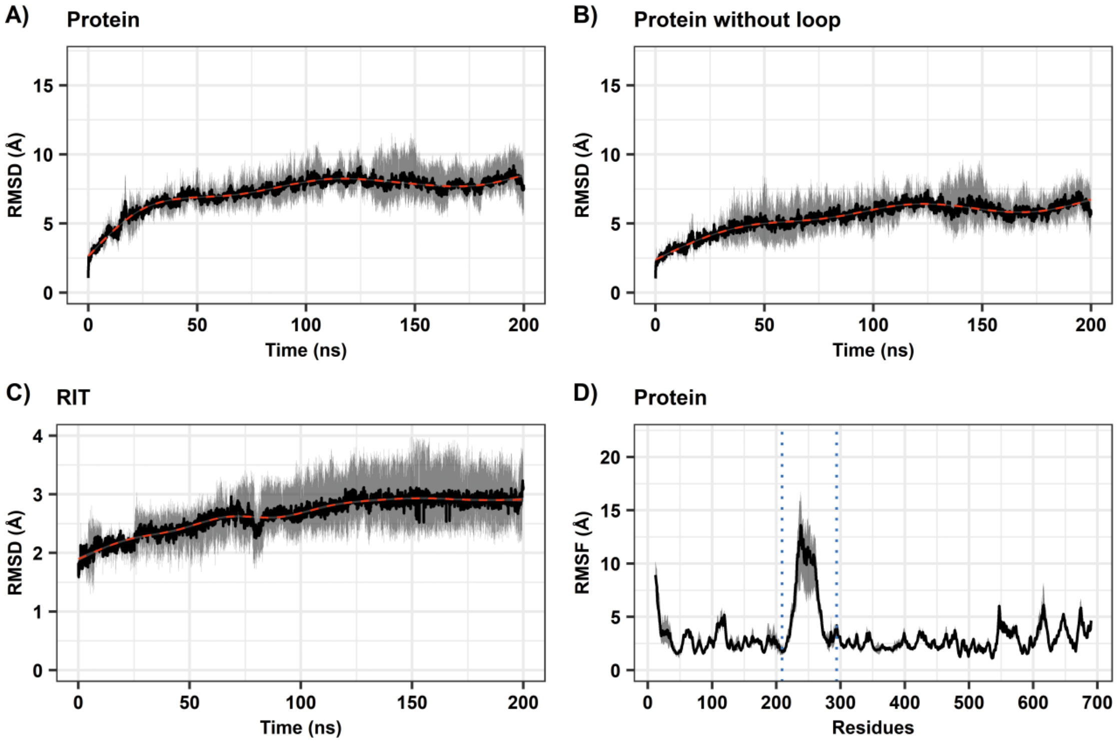

Figure S7. Root mean square deviation and fluctuation of Hsp90β complexed with RIT. A. RMSD of Hsp90β with the DL domain (loop). B. RMSD of the Hsp90β disregarding the DL domain. C. RMSD of RIT. D. RMSF of Hsp90β, the DL region is highlighted. Punctuated red lines represent smoothed averages (black) of fluctuations (gray).
