# Supplementary figures and images for: Structural Characterization of Heat Shock Protein 90β and Molecular Interactions with Geldanamycin and Ritonavir: A Computational Study
Source: Int J Mol Sci. 2024 Aug 12;25(16):8782. doi: 10.3390/ijms25168782 (PMC11354266; doi:10.3390/ijms25168782)

**
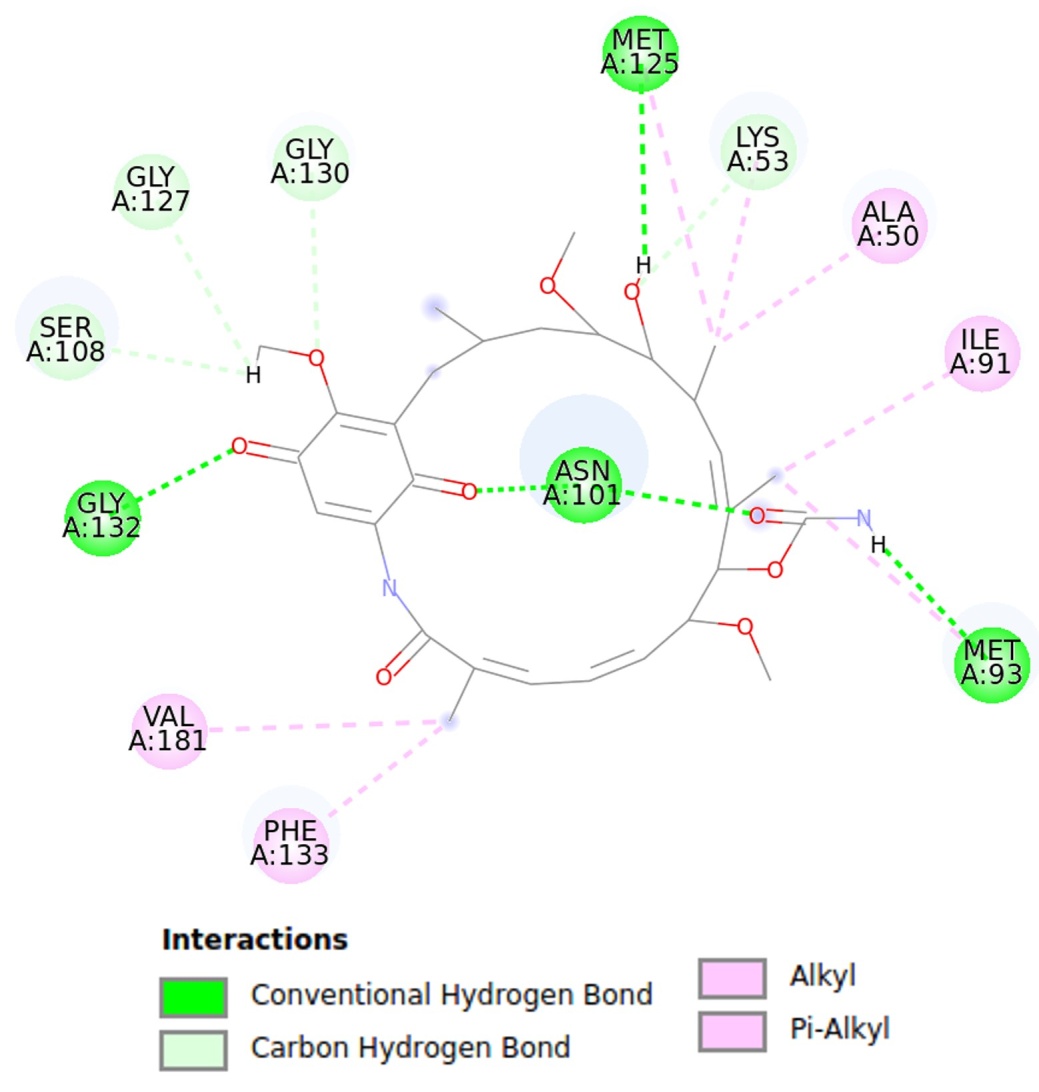
**

**Figure S10**. 2D interaction map of GDM interactions with the best Hsp90β cluster.

Supplement: Supplementary file 1 [file ijms-25-08782-s001.zip › LimaEtAl_SM/FigS10.docx]

**
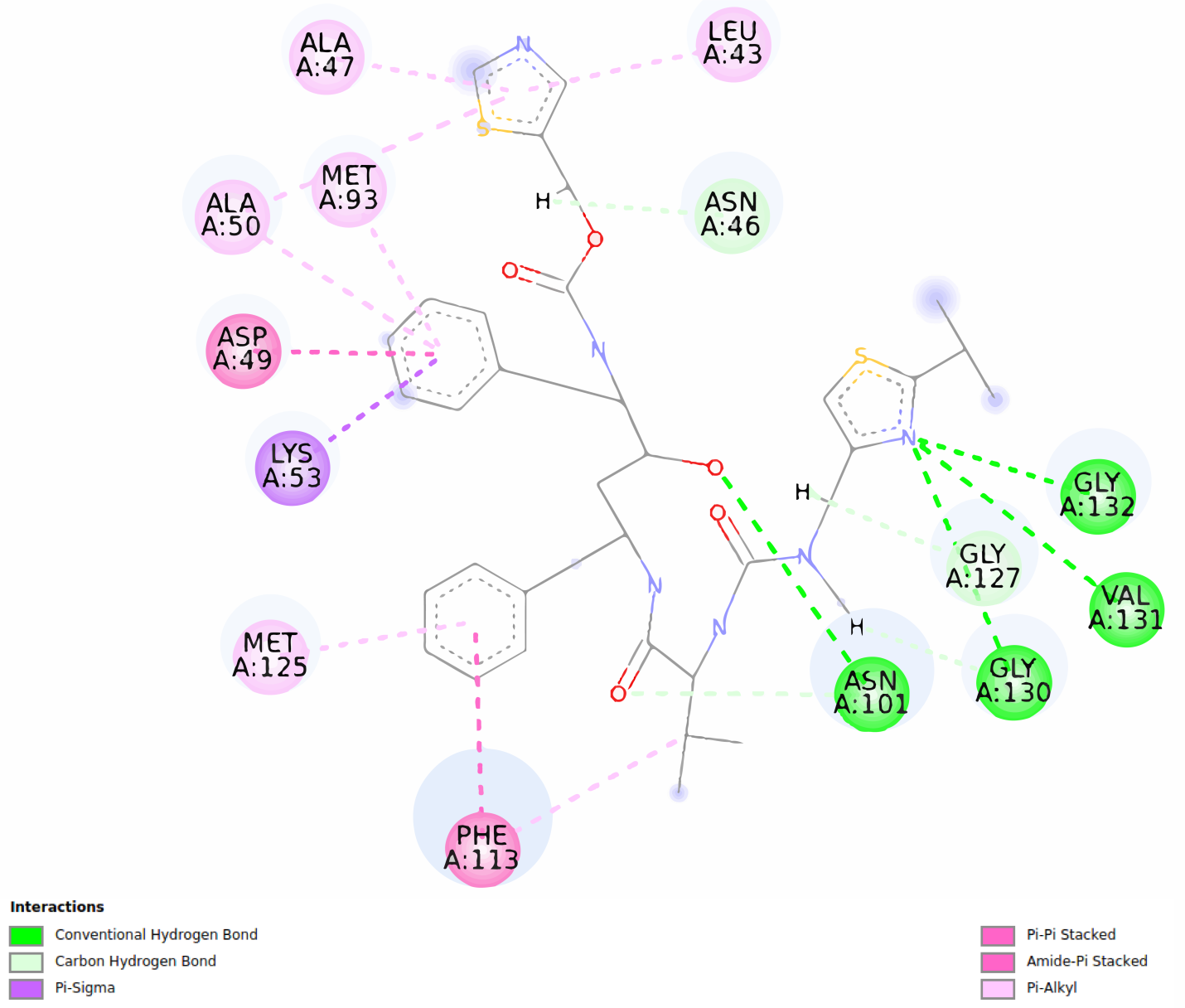
**

**Figure S11**. 2D interaction map of RIT with the best cluster of concatenated DM trajectories of Hsp90β.

Supplement: Supplementary file 1 [file ijms-25-08782-s001.zip › LimaEtAl_SM/FigS11.docx]

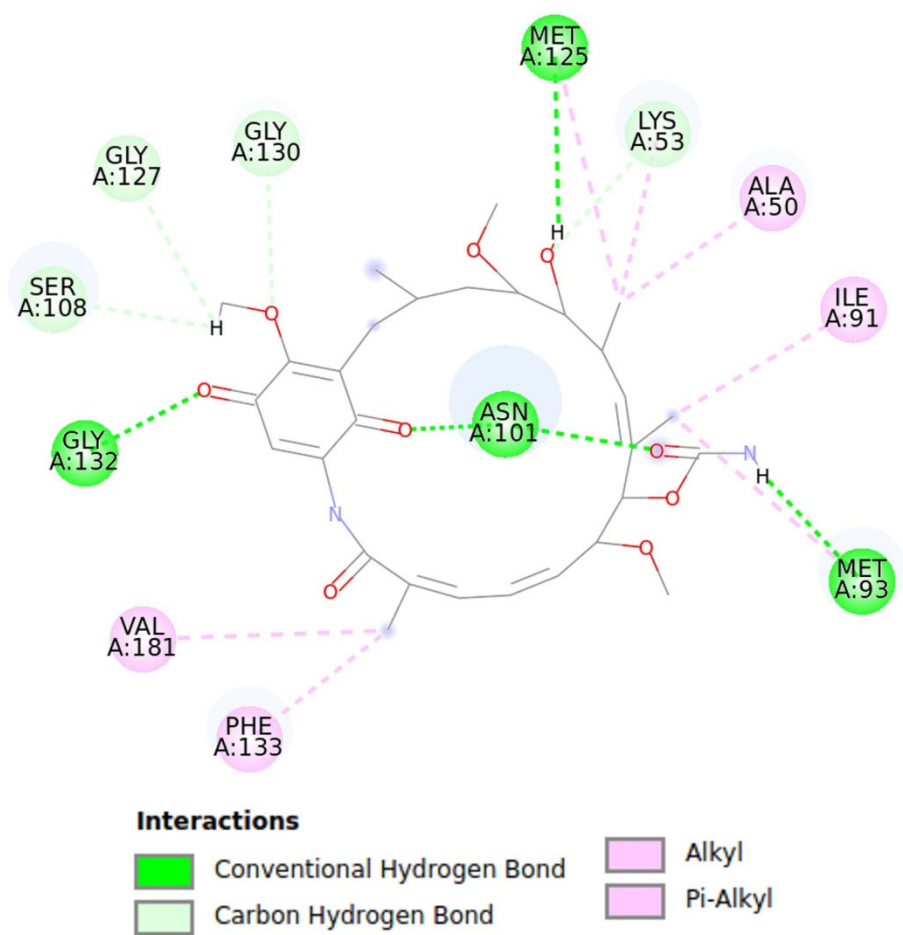

**Figure S10.** 2D interaction map of GDM interactions with the best Hsp90 $\beta$  cluster.

Supplement: Supplementary file 1 [file ijms-25-08782-s001.zip › Figure S10.pdf]
